# Glucagon-Like Peptide‑1 Receptor Agonists Inhibit the Initiation of Toxic Amyloid-β42 Aggregation

**DOI:** 10.1021/jacs.6c01289

**Published:** 2026-05-14

**Authors:** Lucas B. Fallot, Carol A. Anderson, Johnathan R. Pinc, Alisdair Stevenson, Mary Claire Schleck, Ethan Hawryschuk, Owen Z. Li, Julia C. Palchak, Justin R. Toole, Robert W. Kubiak II, Alexander J. Dear, Thomas C. T. Michaels, Ryan Limbocker

**Affiliations:** † Department of Chemical and Biological Science and Engineering, 8531 United States Military Academy, West Point, New York 10996, United States of America; ‡ Photonics Research Center, United States Military Academy, West Point, New York 10996, United States of America; § Department of Biology, Institute of Biochemistry, ETH Zurich, Otto Stern Weg 3, 8093 Zurich, Switzerland; ∥ Bringing Materials to Life Initiative, ETH Zurich, Otto Stern Weg 3, 8093 Zurich, Switzerland

## Abstract

The aggregation of the 42-residue form of the amyloid-β peptide (Aβ_42_) is important in Alzheimer’s disease (AD). Preclinical and clinical findings support that glucagon-like peptide-1 receptor agonists (GLP-1RAs) can protect against neuroinflammation and neurodegeneration with potential therapeutic relevance for AD, but studies of their direct effects on Aβ_42_ are limited. Herein, we investigated five FDA-approved GLP-1RAs, and show semaglutide, tirzepatide, and liraglutide inhibit Aβ_42_ aggregation. Semaglutide and tirzepatide delayed Aβ_42_ aggregation by targeting the primary nucleation microscopic step, with submicromolar IC_50_ values for primary nucleation (*K*_IP_). Liraglutide was highly effective at suppressing primary nucleation with a very low *K*_IP_ value, and it demonstrated an additional modest inhibition of secondary nucleation. Consistent with a dominant effect on primary nucleation, Aβ_42_ formed β-sheet-rich fibrils in the presence of these GLP-1RAs. Aβ_42_ fibrils formed with semaglutide or tirzepatide had morphological properties and templating efficiencies that were similar to unmodified fibrils, while liraglutide significantly reduced fibril maturity, increased fibril tortuosity and length, and attenuated the ability of fibrils to passively self-replicate whether they were formed in the presence of liraglutide or exposed to this GLP-1RA after their formation. These results provide molecular-level insight into how specific GLP-1RAs can selectively target the fundamental steps governing toxic Aβ_42_ aggregation. Further studies are warranted to determine if current or next-generation anti-amyloid GLP-1RAs can delay or prevent AD through multifaceted protective mechanisms, including the direct inhibition of Aβ_42_ aggregation.

## Graphical abstract

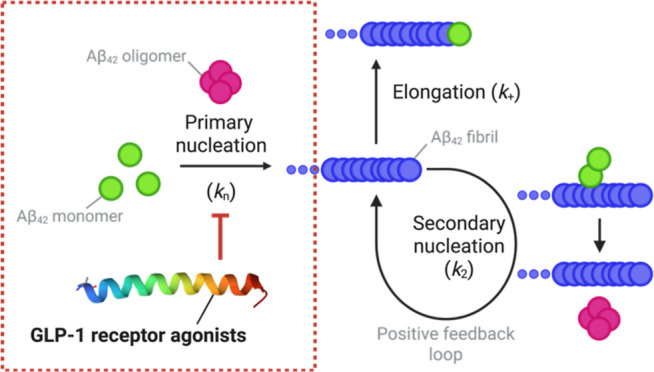

## Introduction

Alzheimer’s disease (AD) is a progressive, fatal neurodegenerative disorder characterized by the aberrant misfolding and aggregation of the amyloid-β peptide (Aβ) and tau protein into amyloid fibrils.^1,2^ Approximately 60-70% of dementia cases are related to AD worldwide, and there are nearly 10 million new cases of dementia every year and with an estimated total of 57 million patients afflicted in 2021.^3^ While the pathophysiology of AD is extremely complex and heterogeneous, this disease is hallmarked by the deposition of Aβ extracellularly into plaques in the brain parenchyma and hyperphosphorylated tau intracellularly in the form of neurofibrillary tangles.^1,2^ The aggregation of these proteins results in multifactorial neuronal dysfunction and progressive cell death, and extensive neuropathological, genetic, and molecular biology studies support the importance of amyloid aggregation in the onset and progression of AD.^1,2,4,5^

Despite extensive efforts to find treatments for AD, there has been limited success in developing disease-modifying therapeutics. Recently FDA-approved monoclonal antibodies can achieve passive immunotherapy to effectively clear Aβ from the AD brain; however, these treatments are not curative, nor are they expected to reverse cognitive and functional deficits.^6^ Anti-amyloid monoclonal antibodies require the early detection of AD and can slow the rate of cognitive and functional decline in such AD patients by ∼30%, but they carry the risk of amyloid-related imaging abnormalities with intracerebral edema, microhemorrhages, or superficial siderosis.^4^ Nonetheless, these treatments help many AD patients and inspire hope that eliminating and/or preventing fibril formation will lead to even better disease-modifying therapeutics for AD. The limited efficacy of any modern AD treatment is likely a result, in large part, of its administration to AD patients at a stage too late in their pathological progression. Since Aβ aggregation can precede clinical manifestations of AD by ∼10–20 years and substantial neuronal loss has occurred by the mild cognitive impairment stage of AD,^5,7^ therapeutic interventions during preclinical stages of disease that are cost-effective and chronically safe are needed to ameliorate disease progression and circumvent the global AD medical emergency.

In the search for such therapeutics, animal and clinical studies have highlighted the potential utility of glucagon-like peptide-1 receptor agonists (GLP-1RAs), which are widely prescribed for Type 2 diabetes mellitus (T2DM), cardiovascular disease, and weight loss, in the treatment of AD potentially by restoring microglial and astrocytic homeostasis, reducing inflammation and oxidative stress, and increasing blood–brain barrier (BBB) integrity and synaptic viability.^8,9^ Numerous studies have linked peripheral and brain insulin resistance to AD risk.^10^ Epidemiological data support that T2DM and cardiovascular disease patients treated with GLP-1RAs have a substantially reduced risk of developing all-cause dementia.^8^ For T2DM patients, treatment with exenatide, liraglutide, dulaglutide, and the dipeptidyl peptidase 4 (DPP-4) inhibitor sitagliptin were associated with a lower risk of AD compared to metformin,^11^ and liraglutide or semaglutide treatment was associated with fewer diagnoses of all-cause dementia.^12^ A target trial emulation study of T2DM patients found that semaglutide can reduce the risk for first-time AD diagnosis.^13^ A multicenter, double-blind, placebo-controlled phase 2b trial for patients with mild to moderate AD syndrome with no diabetes showed liraglutide slowed total gray matter, frontal, temporal, and parietal lobe volume reductions compared to placebo, which was associated with a slower decline in cognitive function.^14^ Preclinical studies show GLP-1RAs may limit the deposition of Aβ and tau in the brain.^15^ Despite these promising preclinical and clinical findings,^8,9^ studies on the mechanisms by which GLP-1RAs interact directly with amyloidogenic proteins are limited.

The gut and neurons in the hindbrain secrete glucagon-like peptide-1 (GLP-1). This peptide hormone is transported to multiple parts of the central nervous system.^8,16^ GLP-1 promotes insulin secretion after a meal in pancreatic β-cells, increases somatostatin release, and attenuates glucagon release from pancreatic α-cells, resulting in slowed gastric emptying, reduced glycemia, and increased satiety.^8,9,17^ GLP-1 is also a neurotransmitter that influences energy intake.^18^ GLP-1 binds to the GLP-1 receptor (GLP-1R), a member of the G protein-coupled receptor (GPCR) family, which is widely expressed in the brain.^19^ As the half-life of GLP-1 is only a few minutes,^20^ GLP-1RAs have been engineered with extended half-life and improved stability, bioavailability, and biological activity.^21^ Half-life has been prolonged by amino acid substitutions that confer resistance to DPP-4 breakdown, the addition of fatty acids that noncovalently bind albumin and extend the time of GLP-1RA circulation, or PEGylation or fusion proteins to increase size and stability.^21^ Exenatide (marketed as Byetta and Bydureon), derived from the saliva of Gila monsters, was FDA-approved in 2005 for treatment of T2DM. Since then, additional GLP-1RAs have been developed and are now widely prescribed for T2DM and chronic weight management, including semaglutide (marketed as Ozempic, Wegovy, and Rybelsus), tirzepatide (marketed as Mounjaro and Zepbound), liraglutide (marketed as Victoza and Saxenda), and dulaglutide (marketed as Trulicity). Sequences and specific chemical modifications for these GLP-1RAs are shown in Figures 1 and S1. Currently, semaglutide, tirzepatide, and liraglutide are FDA-approved for weight management.^22^ Semaglutide, which activates GLP-1R, and tirzepatide, which activates both GLP-1R and the glucose-dependent insulinotropic polypeptide receptor, are widely prescribed medicines.^8^

**Figure 1:**
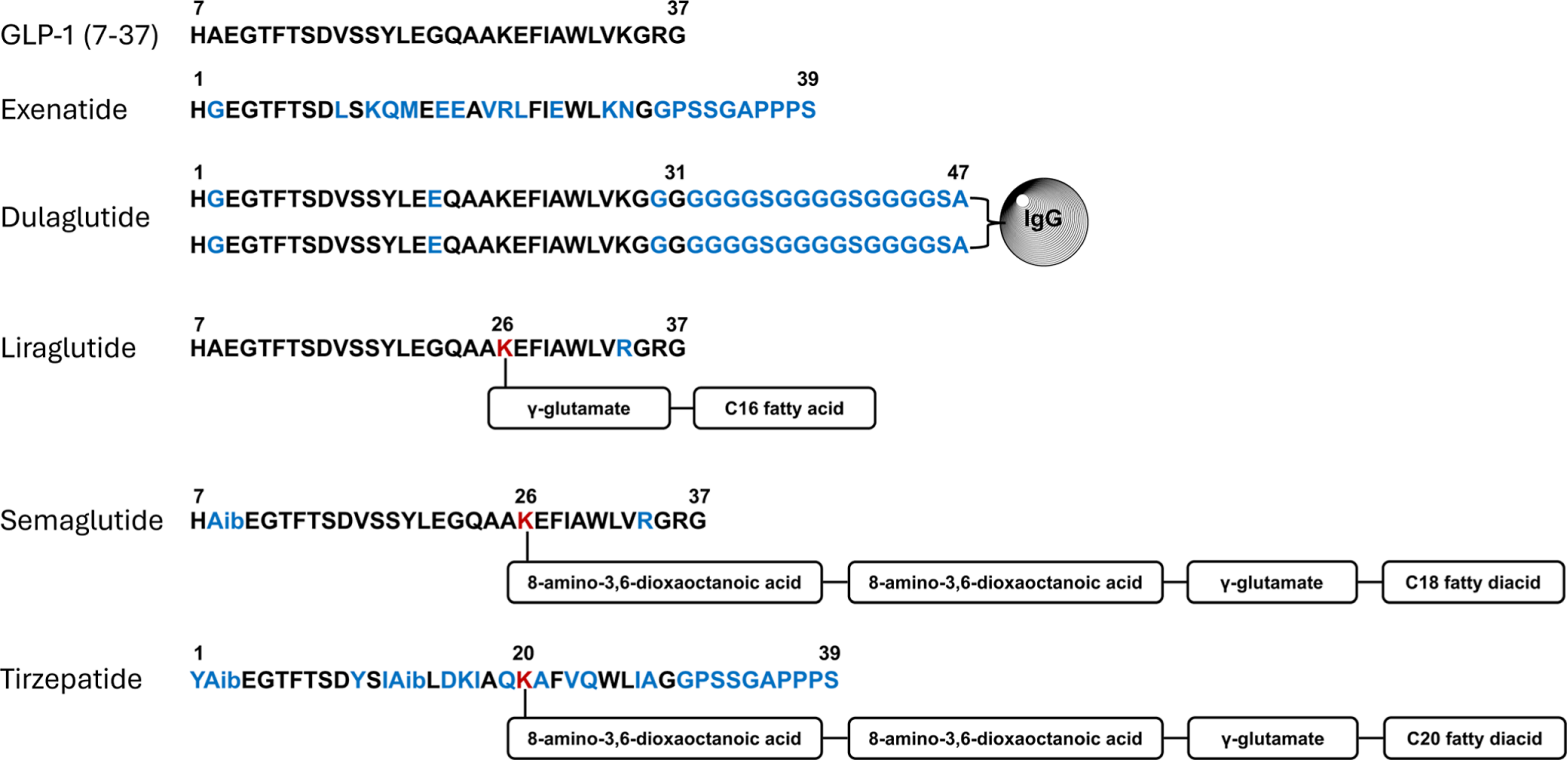
GLP-1­(7–37) and GLP-1RA sequences. Amino acid sequences for GLP-1(7–37) (PDB entry 3IOL, chain B), exenatide (PDB entry 7MLL, chain A), dulaglutide (with a modified IgG4 Fc domain, DrugBank entry DB09045), liraglutide (PDB entry 4APD, chain A), semaglutide (PDB entry 7KI0, chain E), and tirzepatide (PDB entry 7RGP, chain F). Differences in GLP-1RA amino acid sequences relative to GLP-1 are indicated in blue. Aib, 2-aminoisobutyric acid. The lysine residues of liraglutide, semaglutide, and tirzepatide that are modified with linkers are highlighted in red. Expanded structures are shown in Figure S1.

The conversion of proteins from their native state to amyloid fibrils is a heterogeneous and dynamical process that proceeds through a series of intermediates, which can include soluble dimers, trimers, higher-molecular-weight oligomers, and protofibrils, to insoluble fibrils and plaques.^2^ β-sheet content generally increases with aggregate molecular weight.^1,2^ A range of small molecules, antibodies, and chaperones have been shown to inhibit the aggregation of the 42-residue form of Aβ (Aβ_42_), which is the most pathogenic and aggregation-prone form of Aβ,^23^ by targeting specific microscopic processes responsible for aggregate formation and growth. For Aβ, these include primary nucleation, secondary nucleation, and elongation. Molecules can affect one or more of these specific steps,^2,24^ and subtle changes in molecular structure can lead to dramatic differences in their ability to inhibit specific microscopic steps in Aβ_42_ aggregation.^2,25,26^ Considering current evidence supporting the potential use of GLP-1RAs in the treatment of AD and that millions worldwide have or are currently taking GLP-1RAs prior to the onset of AD, we leveraged this rational drug discovery strategy to investigate the impact of five FDA-approved GLP-1RAs on Aβ_42_ aggregation. We show that semaglutide, tirzepatide, and liraglutide significantly inhibit Aβ_42_ aggregation by targeting predominantly primary nucleation reactions. Of these three, liraglutide was the most potent inhibitor of primary nucleation, and it also displayed a relatively smaller, but pertinent ability to inhibit secondary nucleation reactions. The resultant fibrils formed with semaglutide and tirzepatide were structurally similar to Aβ_42_ controls with unchanged templating properties. Liraglutide, however, reduced fibril maturity, increased fibril tortuosity and length, and decreased the ability of Aβ_42_ fibrils to passively self-replicate when present during the fibril formation process or when added to preformed fibrils.

## Results

### Semaglutide, Tirzepatide, and Liraglutide Inhibit Aβ_42_ Aggregation

To study in molecular detail the mechanisms by which GLP-1RAs impact the aggregation of Aβ_42_, we monitored Aβ_42_ fibril formation in vitro using the well-defined thioflavin-T (ThT)-based chemical kinetics assay.^27,28^ Experiments were performed under standardized conditions (20 mM sodium phosphate, 0.2 mM EDTA, 1% v/v DMSO, pH 8.0, 37 °C, quiescent) that enable detailed kinetic analyses.^2,26,29–32^ We first monitored the aggregation of purified monomeric Aβ_42_ at a concentration of 2 μM in the absence and presence of semaglutide and tirzepatide (Figures 2 and S2), liraglutide (Figures 3 and S2), and exenatide and dulaglutide (Figure S3) at ratios of Aβ_42_ to GLP-1RAs of 1:1, 1:5, and 1:10 mol equiv (i.e., 2, 10, and 20 μM of GLP-1RAs). For semaglutide, tirzepatide, and liraglutide, the dose-dependent inhibition of Aβ_42_ aggregation was observed (Figures 2 and 3) with the half-time of Aβ_42_ aggregation increasing by a factor of 1.4, 1.6, and 3.5 in the presence of a 10-fold molar excess of each GLP-1RA, respectively (Figure S4). Exenatide and dulaglutide had a negligible impact on Aβ_42_ aggregation under these conditions (Figure S3).

We confirmed the inhibitory effects of semaglutide, tirzepatide, and liraglutide on Aβ_42_ aggregation using a Congo red fluorescence assay,^29^ similarly finding that all three GLP-1RAs inhibited aggregation. As in the ThT assays, liraglutide again demonstrated greater potency of inhibition relative to semaglutide and tirzepatide (Figure S5). For the highest concentrations of these GLP-1RAs tested, aggregation was not detected in the absence of Aβ_42_ in the Congo red fluorescence (Figure S5d) and ThT (Figure S6) assays. GLP-1RA solubility was checked for the maximum tested concentrations of each GLP-1RA (20 μM, 1% DMSO) using absorbance spectroscopy from 270 to 750 nm. Light scattering can be detected as a sloping, nonzero baseline (e.g., at 600 nm) caused by insoluble particles, which can be confirmed by measuring spectra after centrifugation to remove insoluble species.^33^ Sharp peaks were observed with absorbance maxima near 280 nm (A_280_) caused by tryptophan and tyrosine residues in the GLP-1RAs (Figure S7a) that was negligibly impacted by centrifugation (Figure S7b), supporting that GLP-1RAs are soluble under the tested conditions.

Additives that inhibit the kinetics of amyloid aggregation can do so by suppressing different microscopic processes that govern fibril formation. For Aβ_42_, this includes primary nucleation (the initial, stochastic, and slow formation of oligomers and fibrils directly from Aβ_42_ monomers, with rate constant *k*_n_), monomer-dependent secondary nucleation (the autocatalytic conversion of soluble monomers to oligomers or seeds using the surface of existing fibrils, with rate constant *k*_2_), elongation (the addition of soluble Aβ_42_ to aggregate ends, with rate constant *k*_+_), or a combination of these fundamental steps.^2,33^ From the unseeded kinetic traces, we observed that the lag phase dose-dependently increased for semaglutide, tirzepatide, and liraglutide (Figures 2, 3, and S2). Upon reaching the end of the lag phase, at which time detectable levels of fibrils had formed, however, the slope in the growth phase of the kinetic traces appeared mostly unchanged by semaglutide, tirzepatide, or liraglutide (Figures 2 and 3). Shifted kinetic curves with unaltered slope is consistent with a picture where inhibition of primary nucleation likely dominates the observed macroscopic changes in Aβ_42_ aggregation induced by these GLP-1RAs. Only at higher molar ratios of liraglutide were the slopes of the kinetic traces somewhat attenuated (Figure 3), which suggests that fibril-dependent microscopic steps may be impacted under those conditions.

### Kinetic Analyses Show Semaglutide, Tirzepatide, and Liraglutide Predominantly Suppress the Rate Constant for Primary Nucleation

We next conducted a quantitative analysis of the impacts of GLP-1RAs on Aβ_42_ aggregation by comparing the experimental kinetic data from the unseeded aggregation experiments with kinetic curves obtained by solving analytically the rate laws of aggregation kinetics, as described in detail previously.^2,27,30,34^ This enables the macroscopic time-dependent proliferation of aggregates to be expressed as a function of the rate constants for each of the aforementioned contributing microscopic processes. First, the rate constants for primary nucleation (*k*_*n*_), secondary nucleation (*k*_2_), and elongation (*k*_+_) were determined for Aβ_42_ in the absence of GLP-1RAs across the unseeded, 5% seeded, and 25% seeded assays (Figure S8) and maintained constant in all subsequent fits. We then globally fit the tested concentrations of semaglutide and tirzepatide to mathematical models where only primary nucleation, only secondary nucleation, or only elongation was perturbed. Fitting the unseeded and 5% seeded kinetic traces by modifying globally just *k*_2_ or *k*_+_ resulted in clear misfits, whereas fitting globally for *k*_*n*_ recapitulated well the experimental data (Figure S9). Next, we fit just the unseeded data as above, obtaining high-quality fits when fitting globally for the attenuation of only primary nucleation by semaglutide and tirzepatide (Figure 2a), whereas fitting for only secondary nucleation or elongation yielded lower quality fits (Figure 2b,c). These kinetic models are consistent with the above observations on changes to the lag and growth phases of aggregation and support that the predominant, although not necessarily exclusive, mechanism of action for semaglutide and tirzepatide is the targeting of primary nucleation. The IC_50_ value for primary nucleation (*K*_IP_), which describes the concentration of inhibitor at which primary nucleation is half-maximally inhibited and lower values indicate more potent inhibition, was 0.422 and 0.325 μM for semaglutide and tirzepatide, respectively (Figure 2a). According to the model, these values are also dissociation constants, assuming the equilibrium reversible binding approximation is accurate.

Following the same procedures for the liraglutide data, we found poor fits when fitting globally for only the attenuation of primary nucleation (Figure 3a), secondary nucleation (Figure 3b), or elongation (Figure 3c). Accounting for the observed flattening of the slope during the growth phase of aggregation upon the use of higher concentrations of liraglutide, the data suggest that this GLP-1RA impacted more than just primary nucleation. We therefore fit the data for effects on both primary and secondary nucleation, yielding excellent fits (Figure 3d) with *K*_IP_ = 8.35 × 10^–7^ μM and *K*_IS_ = 33.2 μΜ (IC_50_ values for primary and secondary nucleation, respectively). The subpicomolar *K*_IP_ and supermicromolar *K*_IS_ values support that liraglutide mainly targets primary nucleation, but its impact on secondary nucleation is important. In summary, semaglutide, tirzepatide, and liraglutide all inhibit Aβ_42_ aggregation by perturbing predominantly the primary nucleation microscopic step, and the potency of this effect assigned from the kinetic fitting procedure scales from submicromolar *K*_IP_ values for semaglutide and tirzepatide to a subpicomolar *K*_IP_ for liraglutide.

**Figure 2:**
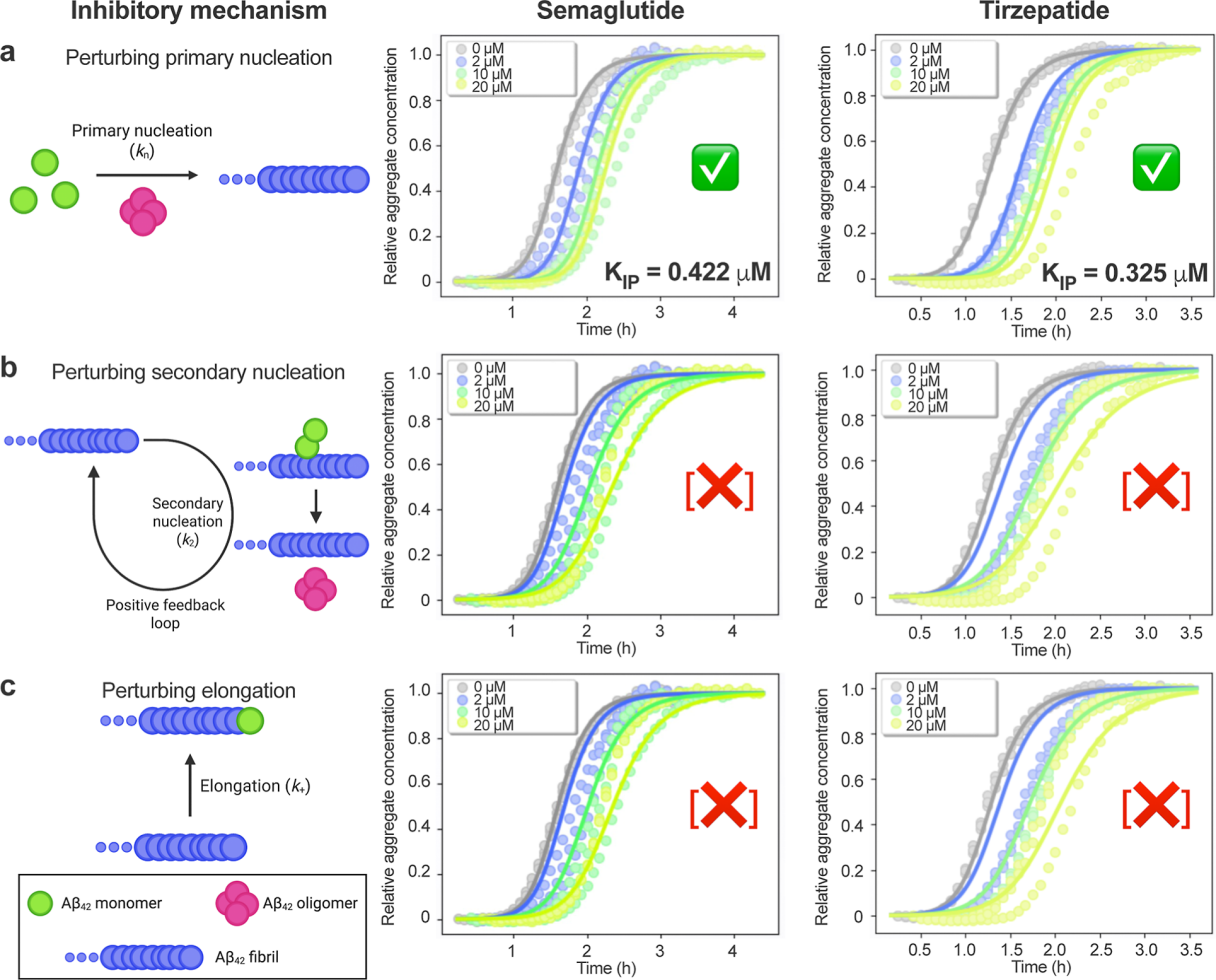
Semaglutide and tirzepatide inhibit Aβ_42_ aggregation by suppressing predominantly primary nucleation reactions. Mechanism of action determination for GLP-1RAs by global fitting Aβ_42_ aggregation kinetics with different concentrations of each inhibitor. Experiments were performed using 2 μM Aβ_42_ (in 20 mM sodium phosphate, 0.2 mM EDTA, 1% v/v DMSO, pH 8.0, quiescent) in the absence (gray) or presence of 2 (blue), 10 (green), or 20 μM (yellow) concentrations of semaglutide (left) or tirzepatide (right). Solid lines indicate theoretical predictions based on kinetic fitting (see Results). During fitting, the rate constants for primary nucleation (*k*_n_), secondary nucleation (*k*_2_), and elongation (*k*_+_) were determined for Aβ_42_ alone (Figure S8), and then held as global constants while fitting for perturbations to only (a) primary nucleation, (b) secondary nucleation, or (c) elongation across the different inhibitor concentrations. Green checks indicate high-quality fits with associated IC_50_ values for primary nucleation (*K*_IP_). [X] symbols indicate lower quality fits. We also simultaneously fit the unseeded and 5% seeded data in the above manner, which support that semaglutide and tirzepatide inhibited aggregation mainly by targeting primary nucleation, rather than secondary nucleation or elongation (Figure S9). Illustrations created with BioRender.com. Three technical replicates are shown for each condition.

### Seeded Aggregation Experiments Support a Primary Pathway-Dominated Mechanism of Action for Semaglutide, Tirzepatide, and Liraglutide

To further assess the impact of semaglutide, tirzepatide, and liraglutide on secondary processes, we next monitored the aggregation kinetics of Aβ_42_ in the presence of preformed fibrils that act as seeds that immediately accelerate aggregate growth and multiplication. Under these conditions, Aβ_42_ aggregation follows an efficient positive feedback mechanism, in which fibril surfaces act as catalytic sites for secondary nucleation. This process significantly enhances the conversion of soluble monomeric Aβ_42_ into oligomeric species, which can subsequently grow and elongate to form new fibrils. Surface-catalyzed secondary nucleation has been shown to dominate the in vitro aggregation of Aβ_42_ once primary nucleation produces a low but critical concentration of aggregates.^26^ The addition of seed fibrils at the start of the reaction, therefore, markedly accelerates fibril formation by promoting *k*_2_ and *k*_+_. Under these conditions, *k*_n_ is no longer rate-limiting and is negligible in the overall rate of Aβ_42_ fibril formation.^2,26^

**Figure 3:**
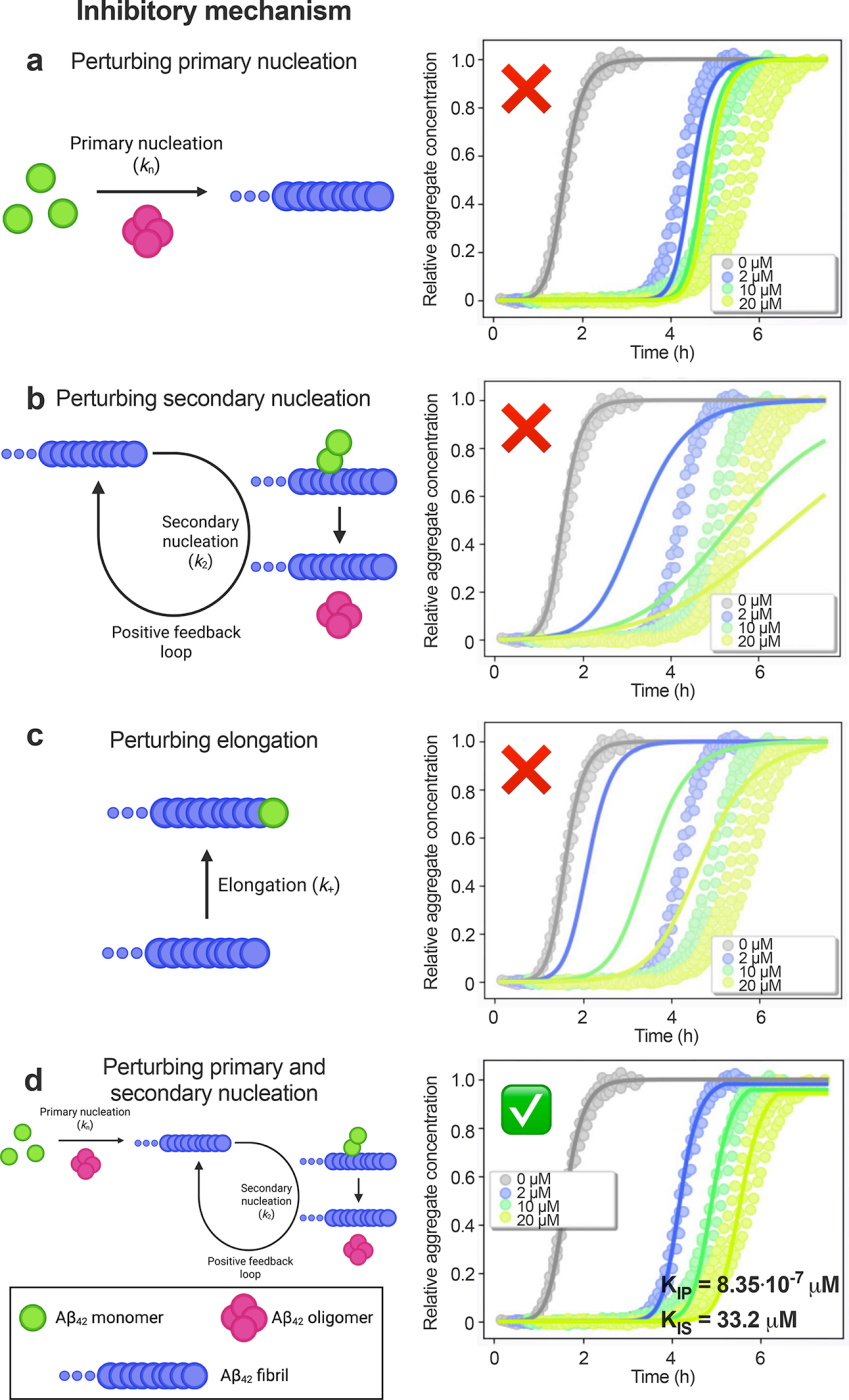
Liraglutide strongly inhibits primary nucleation and modestly suppresses secondary nucleation. Experiments were performed using 2 μM Aβ_42_ (in 20 mM sodium phosphate, 0.2 mM EDTA, 1% v/v DMSO, pH 8.0, quiescent) in the absence (gray) or presence of 2 (blue), 10 (green), or 20 μM (yellow) concentrations of liraglutide. The same analytical procedures were followed as in Figure 2, while fitting for perturbations to only (a) primary nucleation, (b) secondary nucleation, or (c) elongation across the different inhibitor concentrations, or fitting for both primary and secondary nucleation (d). Green check indicates high-quality fits with associated IC_50_ values for primary nucleation (*K*_IP_) and secondary nucleation (*K*_IS_). Red X marks indicate misfits. Illustrations created with BioRender.com. Three technical replicates are shown for each condition.

We monitored the aggregation of 2 μM monomeric Aβ_42_ in the presence of 5% fibril seeds or 25% fibril seeds (in monomer equivalents) and GLP-1RAs at the same molar ratios of Aβ_42_ to GLP-1RAs as above. With 5% seeds, primary nucleation is not rate-limiting. With 25% seeds, elongation of the preformed fibrils has been found to dominate aggregate growth, and under these conditions, neither primary nor secondary nucleation processes are rate-limiting.^2,26^ For both the low and high-seeded assays, aggregation traces were minimally impacted for semaglutide and tirzepatide (Figure S2), supporting that neither of these GLP-1RAs exert an overt impact on secondary nucleation or elongation. The presence of liraglutide under analogous conditions, however, did inhibit Aβ_42_ aggregation in both the low and high-seeded assays (Figure S2). Fitting the low-seeded data with only a perturbation to secondary nucleation yielded decent fits, with *K*_IS_ = 2.61 μΜ (Figure S10a). Similarly, fitting the high-seeded data for only a perturbation to only elongation or only secondary nucleation (Figure S10b) yielded decent fits, with *K*_IE_ = 2.61 μΜ and *K*_IS_ = 2.60 μΜ, respectively. We note that liraglutide, more so than semaglutide or tirzepatide, interacted with ThT at the start of all unseeded and seeded aggregation reactions, as evidenced by a dose-dependent increase in ThT fluorescence signal when the plate was first measured, which quickly decreased to a stable baseline after ∼0.5 h (Figure S2c). This phenomenon was beyond the normal thermal equilibration of ThT, as it changes from ice cold to 37 °C under these experimental conditions, as observed in the various samples lacking GLP-1RAs (Figure S2). These effects were facile to account for in an unseeded assay, where there was plenty of time for the baseline to be established, but more difficult to model in seeded assays with liraglutide where fibril formation occurs within the first 0.5 h of the aggregation reaction. Considering this and the lower quality of the fits (Figure S10), the specific *K*_IE_ or *K*_IS_ values should be interpreted with caution; nonetheless, the IC_50_ values in the seeded assays are within approximately an order of magnitude of *K*_IS_ determined from the unseeded fits, and these *K*_IE_ or *K*_IS_ values are several orders of magnitude higher than the *K*_IP_ value from the unseeded assay. Additionally, we leverage complementary biophysical measurements described below to assess whether the effect on secondary processes by liraglutide is driven by a decrease in *k*_2_ or *k*_+_.

### Structural Characterization of Aβ_42_ Oligomers Exposed to GLP-1RAs

While semaglutide, tirzepatide, and liraglutide inhibited Aβ_42_ aggregation with differing potencies, these three GLP-1RAs acted predominantly by suppressing the primary nucleation microscopic step (Figures 2 and 3). Inhibition of primary nucleation requires GLP-1RAs to interact with monomeric and/or oligomeric Aβ_42_. Litus et al. observed that monomeric Aβ_40_ and Aβ_42_ can directly bind to GLP-1(7–37), semaglutide, exenatide, and liraglutide, with liraglutide showing the highest affinity (*K*_D_ of 42–60 nM).^35^ We therefore next sought to determine if GLP-1RAs can impact the structures of stabilized oligomeric Aβ_42_. Using Lambert’s protocol, Aβ-derived diffusible ligands (ADDLs) were prepared into a relatively homogeneous population of Aβ_42_ oligomers with hydrodynamic radii peaking at ∼38 nm (Figure S11), consistent with past studies.^30^ Oligomers at a concentration of 10 μM were then incubated in the absence or presence of 1:1, 1:2, and 1:4 molar ratios of Aβ_42_ to each GLP-1RA (with 2% v/v DMSO) for 1 h at 37 °C. We observed by dynamic light scattering (DLS) that semaglutide and tirzepatide had negligible effects on the size of Aβ_42_ oligomers in solution, whereas liraglutide dose-dependently decreased the observed hydrodynamic radii of the aggregates (Figure S11), suggesting this GLP-1RA, uniquely, can interact with Aβ_42_ oligomers.

### Structural Characterization of Aβ_42_ Fibrils Formed in the Presence of GLP-1RAs

We next sought to determine the impact of GLP-1RAs on the structures of fibrils formed in the presence of semaglutide, tirzepatide, and liraglutide. Samples were incubated as in the kinetic experiments, but in the absence of ThT. Once all samples had finished aggregating, as assessed using tracer samples containing ThT in adjacent wells, aliquots were deposited onto mica grids functionalized with APTES. APTES imparts a positive charge on the mica substrate at near-neutral pH values, thereby favoring the adsorption of negatively charged Aβ_42_ and GLP-1RA molecules.^29^ Samples were imaged by means of high-resolution, phase-controlled atomic force microscopy (AFM) to characterize the morphology of the aggregates formed in the absence or presence of a 1:10 molar ratio of Aβ_42_ to GLP-1RA (i.e., 2 μM Aβ_42_ to 20 μM GLP-1RA).

In the absence of GLP-1RAs, Aβ_42_ fibrils had mean cross-sectional heights of 4.4 ± 0.3 nm (mean ± s.e.m., Figure 4a). The presence of 20 μM of each of these GLP-1RAs during the fibril formation process resulted in measured fibril heights of 4.4 ± 0.2 nm for semaglutide (*P* = 0.9779), 3.8 ± 0.2 nm for tirzepatide (*P* = 0.0641), and 2.9 ± 0.1 nm for liraglutide (*P* < 0.0001, one-way ANOVA followed by Dunnett’s postcomparison test) (Figure 4b–e). Fibrils formed in the presence of liraglutide therefore displayed a prominent decrease in maturity, alongside a significant increase in tortuosity (where $\tau =\frac{actual\;path\;length}{straight\;line\;distance}$ , from 1.028 ± 0.003 in its absence to 1.073 ± 0.012 in its presence, *P* = 0.0001) and greater length (from 528 ± 32 nm in its absence to 1330 ± 131 nm in its presence, *P* < 0.0001) (Figure 4e). The inclusion of semaglutide or tirzepatide during the fibril formation process did not significantly alter the morphology of the Aβ_42_ fibrils, as assessed by cross-sectional height, tortuosity, and length (Figure 4e).

The comparison of the length distributions of the fibrillar aggregates showed that only fibrils formed in the presence of liraglutide were significantly different in size (longer) relative to unmodified fibrils (Figure 4e). A decrease in the frequency of secondary nucleation events would result in the formation of elongated fibrils. Specifically, in a system dominated by secondary nucleation, fibril length is expected to scale with $mean\;length=\sqrt{\frac{k_{+}}{k_{2}}}$ .^36^ The observed ∼2.5-fold increase in fibril length with 20 μM liraglutide therefore supports the inhibition of secondary nucleation by liraglutide. This change in length equates to a ∼6.25-fold drop in secondary nucleation. As $K_{IS}=\frac{c_{d}}{k_{2}}$ (where *c*_d_ is the concentration of inhibitor), these results correspond to a *K*_IS_ of ∼4 μM. This is lower than the *K*_IS_ determined from the unseeded assay (33.2 μM, Figure 3) and slightly higher than the *K*_IS_ from the seeded experiments (2.6 μM, Figure S10). These different methods of determining *K*_IS_ are in good agreement, given they are within about one order of magnitude. The appearance of thinner fibrils upon secondary nucleation inhibition has also been observed for Brichos, which is a potent inhibitor of secondary nucleation in Aβ_42_ aggregation.^37^ Secondary nucleation sites were recently shown to be defects in the Aβ_42_ fibril surface, and the higher-order assembly of filaments was postulated to be anchored by these defects.^38^ These results support that inhibiting secondary nucleation reactions that take place at defect sites in fibrils may suppress the bundling of filaments into fibrils, with the final result of fewer filaments per fibril.

In addition to Aβ_42_ fibrils being significantly less mature when formed in the presence of liraglutide, small stabilized species were observed for liraglutide at the end of the aggregation reaction (Figure 4d), whereas predominantly only fibrillar species were observed upon the incubation of Aβ_42_ with semaglutide and tirzepatide (Figure 4b,c). GLP-1RAs incubated under identical conditions as above, but in the absence of Aβ_42_, were also measured by AFM (Figure 4f–h). As in the case of liraglutide with Aβ_42_, small stabilized species were similarly observed for liraglutide under these conditions (Figure 4h). This suggests liraglutide can form stabilized species that can persist in the Aβ_42_ aggregation reaction over the ∼6 h incubation period used herein. We note that we performed our AFM imaging in air, and these measurements can be susceptible to drying or surface mass transport artifacts, as well as differences in GLP-1RA deposition on APTES-functionalized mica. To further assess this, we also performed DLS measurements at room temperature in solution, finding that liraglutide has a hydrodynamic radius that peaks at ∼3.4 nm under these solution conditions, while tirzepatide is ∼3.9 nm and semaglutide is ∼59 nm (Figure S11). Similar DLS results were obtained at 37 °C (Figure S12).

**Figure 4:**
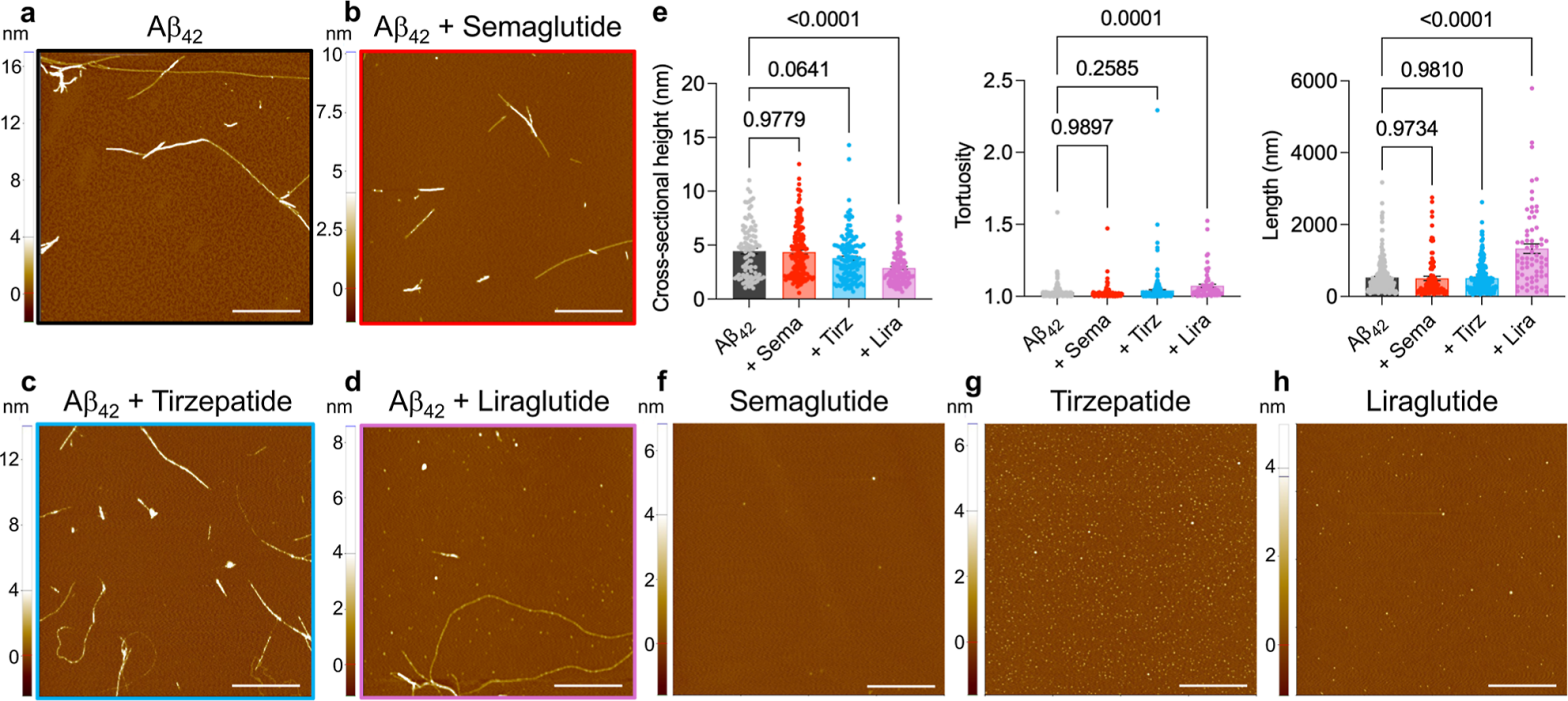
AFM analysis of Aβ_42_ fibril morphology upon formation in the presence of semaglutide, tirzepatide, or liraglutide. Samples were deposited at the end of the aggregation reaction for Aβ_42_ fibrils formed in the absence (a) or presence of a 10-fold molar excess concentration of semaglutide (Sema), tirzepatide (Tirz), or liraglutide (Lira) (b-d). (e) Quantification of fibril cross-sectional heights (left), tortuosity (middle), and length (right). Data were analyzed by one-way ANOVA with Dunnett’s postcomparison test relative to unmodified Aβ_42_ fibrils. (f–h) GLP-1RAs incubated under the same conditions as (a–d), but in the absence of Aβ_42_. Scale bars, 1 μm.

It has been reported that GLP-1RAs can form micelles, oligomers, and fibrils.^39^ The addition of lipidated moieties to therapeutic peptides (e.g., palmitic acid in liraglutide, C18 fatty diacid in semaglutide, and C20 fatty diacid in tirzepatide) increases their tendency to self-assemble and also to bind human serum albumin, which confers resistance to enzymatic degradation and slows renal clearance.^40^ Although our above Congo red and ThT experiments performed in the absence of Aβ_42_ did not show detectable fibril formation over time in these tinctorial assays (Figures S5d and S6), we further explored this possibility using AFM. For semaglutide, tirzepatide, or liraglutide incubated in the absence of Aβ_42_ (Figure 4f–h), we imaged the surfaces of these samples extensively across different regions and found no fibrillar species in the semaglutide or the tirzepatide samples. For liraglutide, however, a total of two fibrillar species were observed after exhaustive imaging, suggesting this is a rare, but possible phenomenon (Figure S13).

Given the observed differences in fibril cross-sectional height, we next assessed the secondary structures of fibrils formed in the presence of GLP-1RAs using FTIR spectroscopy. Samples were prepared as in the AFM measurements, after which time they were centrifuged and concentrated for FTIR analysis (see Methods). Aβ_42_ fibrils demonstrated rich intermolecular β-sheet content (Figure 5), consistent with expectations for amyloid fibrils. The presence of semaglutide, tirzepatide, and liraglutide during the fibril formation process did not overtly change the measured FTIR spectra (Figure 5a). Analysis of second derivatives of normalized FTIR spectra supports that the structures of the fibrils were similar with and without GLP-1RAs (Figure 5b). A small increase in α-helical content was appreciated for tirzepatide, as evidenced by an increase in absorbance at 1654 cm^–1^ (Figure 5).

### Assessment of Seeding Capacity for Fibrils Formed in the Presence of GLP-1RAs

Given the AFM and FTIR results, we next sought to determine whether fibrillar structures formed when Aβ_42_ aggregates in the presence of GLP-1RAs would differ in their ability to template further Aβ_42_ aggregation. We also assessed whether incubating GLP-1RAs with preformed Aβ_42_ fibrils would affect their templating abilities. First, aggregation experiments were performed without ThT in the absence or presence of a 1:10 molar ratio of Aβ_42_ to GLP-1RAs. After ∼6 h at 37 °C, when all samples had finished aggregating (according to corresponding tracer samples in adjacent wells containing ThT), insoluble aggregates formed under the various conditions were isolated by two cycles of centrifugation and resuspension in the same 20 mM phosphate buffer used above. Fibril stocks were thus generated for unmodified fibrils or fibrils formed in the presence of a 1:10 molar ratio of Aβ_42_ to semaglutide, tirzepatide, or liraglutide. Aggregation reactions were then prepared using 2 μM monomeric Aβ_42_ supplemented with 2%, 5%, or 10% (in monomer equivalents) preformed fibrils from each stock (Figure 6a). Fibrils formed in the presence of semaglutide or tirzepatide demonstrated comparable seeding capacities to unmodified Aβ_42_ fibrils, while those formed in the presence of liraglutide showed a modest reduction in seeding capacity (Figure 6a). Of note, the fibril stock preparation procedure was followed for mixtures of GLP-1RAs at the highest tested concentrations, but in the absence of Aβ_42_. Following centrifugation of these mixtures and their resuspension in phosphate buffer, identical aggregation reactions were prepared as above. No difference in Aβ_42_ aggregation was appreciated under these conditions (Figure S14), indicating the observed reduction in seeding capacity for liraglutide is not caused by the presence of residual free GLP-1RA carried over to the final aggregation reaction.

**Figure 5:**
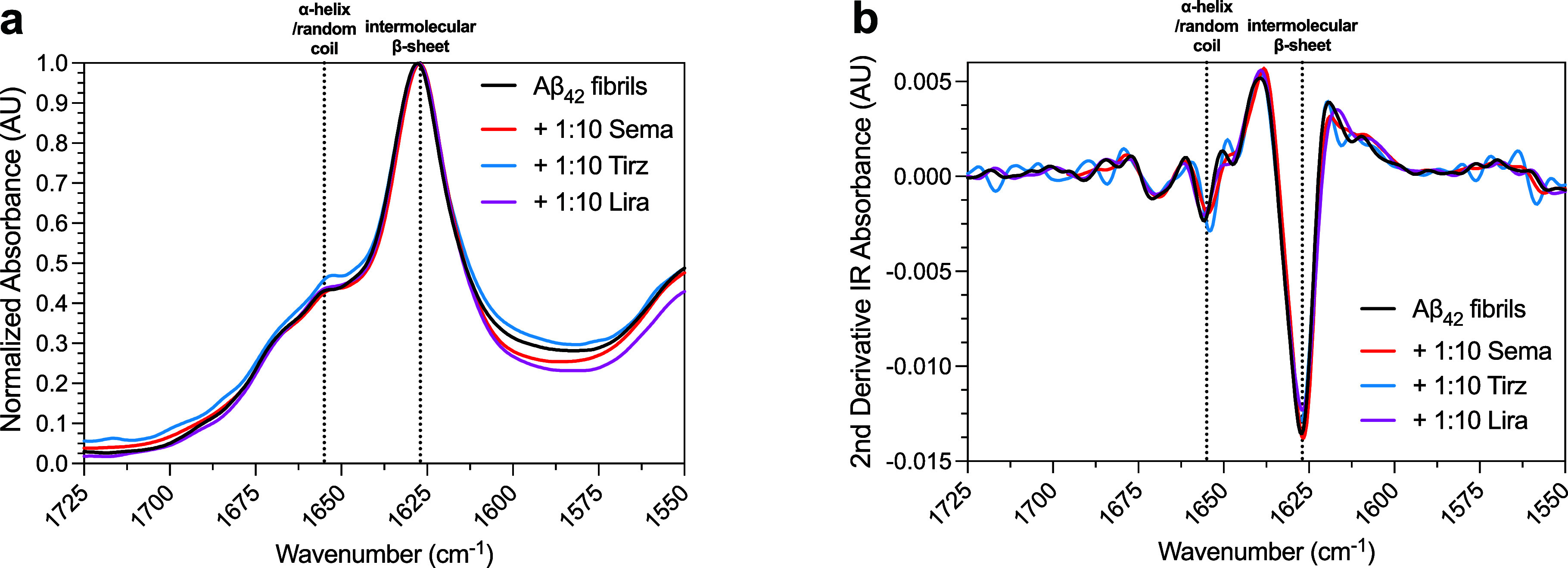
Secondary structure analysis for fibrils formed in the presence of GLP-1RAs. FTIR spectra (a) and associated 2nd derivatives (b) for Aβ_42_ aggregated in the absence (black) or presence of a 10-fold molar excess of semaglutide (red), tirzepatide (blue), or liraglutide (purple). Data are representative of two independent experiments.

### Assessment of Seeding Capacity for Fibrils Exposed to GLP-1RAs after Their Formation

Preformed Aβ_42_ fibrils were similarly incubated without ThT in the absence or presence of a 1:10 molar ratio of Aβ_42_ to GLP-1RAs. After the same ∼6 h period at 37 °C, insoluble aggregates were again isolated by centrifugation followed by resuspension in 20 mM phosphate buffer as above. New fibril stocks were thus generated for unmodified fibrils or fibrils exposed after their formation to a 1:10 molar ratio of Aβ_42_ to semaglutide, tirzepatide, or liraglutide. Aggregation reactions were again prepared using 2 μM monomeric Aβ_42_ supplemented with 2%, 5%, or 10% (in monomer equivalents) preformed fibrils from these stocks (Figure 6b). Fibrils exposed after their formation to semaglutide or tirzepatide again showed equivalent seeding capacities to unmodified Aβ_42_ fibrils, while those incubated with liraglutide demonstrated a pronounced reduction in seeding capacity (Figure 6b).

**Figure 6:**
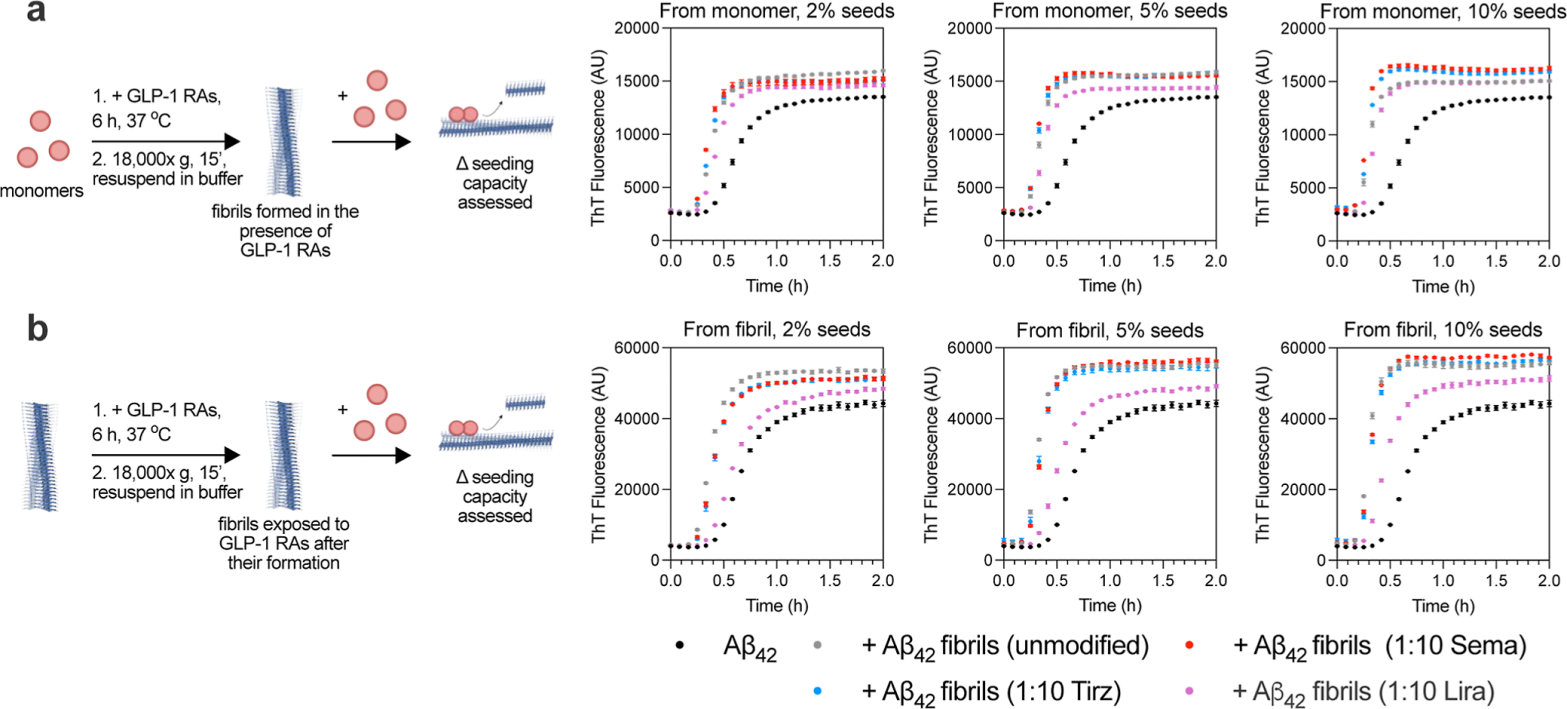
Liraglutide reduces the seeding capacity of Aβ_42_ fibrils. (a) 2 μM Aβ_42_ was aggregated to completion in the absence (black) or presence of a 1:10 molar ratio of Aβ_42_ to semaglutide (red), tirzepatide (blue), or liraglutide (purple) (samples denoted “from monomer”). Solutions were centrifuged to pellet insoluble material, followed by resuspension in phosphate buffer for two cycles. Unmodified or GLP-1RA-modified fibril stocks were then added at 2% (left), 5% (middle), or 10% (right) concentrations to seed the aggregation of 2 μM Aβ_42_. (b) GLP-1RAs were added to preformed fibrils under the same conditions (samples denoted “from fibril”). Residual unbound GLP-1RA was not carried over during the stock formation process (Figure S14). Additional experiments with 1:1 and 1:10 molar ratios of Aβ_42_ to GLP-1RA are shown in Figure S15. Error bars indicate s.e.m. of three technical replicates. Illustrations created with BioRender.com.

We repeated the above procedures to form the various fibril stocks in the absence or presence of 1:1 and 1:10 molar ratios of Aβ_42_ to GLP-1RAs, observing highly similar results. The addition of semaglutide or tirzepatide at the start of the aggregation or their addition to preformed fibrils did not alter the seeding capacity of Aβ_42_ fibrils (Figure S15). Liraglutide caused a modest, dose-dependent reduction in seeding capacity when present during the fibril formation process and a more prominent, dose-dependent reduction when added to preformed fibrils (Figure S15). Control samples with the highest tested concentrations of GLP-1RAs (20 μM) without Aβ_42_ treated in the same way as the experimental conditions had no impact on seeding capacity, confirming residual free GLP-1RA was not appreciably carried over from the various fibril stocks (Figure S16). Both seeding capacity experiments (Figures 6 and S15) show that liraglutide had a more pronounced impact on seeding capacity when added to preformed fibrils than when it was added at the start of the fibril formation process.

Using the ThT assay, we also checked if 2 μM preformed Aβ_42_ fibrils incubated with a 1:10 molar ratio of semaglutide, tirzepatide, and liraglutide could induce the disaggregation of Aβ_42_ fibrils. The ThT signal remained stable over time, with no relevant decreases, supporting that these GLP-1RAs do not disaggregate preformed Aβ_42_ fibrils (Figure S17). The observation that liraglutide reduces Aβ_42_ seeding capacity when added to its preformed fibrils suggests that this GLP-1RA, uniquely, may interact with surfaces on Aβ_42_ fibrils to block its autocatalytic sites. These data are consistent with the aggregation kinetic experiments, where only liraglutide showed inhibitory activity in the seeded aggregation assays using unmodified Aβ_42_ fibril seeds (Figure S2). Moreover, the reduction in seeding capacity for fibrils exposed after their formation to liraglutide became smaller as the percentage of fibril seeds increased (Figure 6b). As elongation is favored over secondary nucleation as the seed concentration increases, these data further complement the kinetic (Figure 3), AFM (Figure 4) and DLS results on oligomers (Figure S11), which collectively support that liraglutide targets secondary nucleation.

### GLP-1RAs do Not Appreciably Copellet with Aβ_42_ Fibrils

Our kinetic models support that semaglutide and tirzepatide, which predominantly target primary nucleation, most likely interact substantially with soluble on-pathway aggregation intermediates (i.e., oligomers produced by primary nucleation) rather than the soluble monomers or insoluble mature fibrils, which is supported by an absence of their impacts to seeded Aβ_42_ aggregation and the fact that fibrils formed in their presence were morphologically similar to unmodified Aβ_42_ fibrils. Alternatively, these peptides could be preferentially binding to nonprotein interfaces in the system (i.e., air–water interface or the surface of the reaction vessel) that are generally the favorable sites for primary nucleation. Liraglutide, however, could be interacting with both soluble and insoluble Aβ_42_ species, resulting in a marked decrease in primary nucleation, a smaller decrease in secondary nucleation, lowered fibril width, increased fibril length, and attenuated fibril templating ability. FTIR spectra for fibrils formed in the presence of all three GLP-1RAs were highly similar, consistent with observations that all three compounds target predominantly primary nucleation. We next sought to assess if GLP-1RAs remain in the soluble or insoluble fraction during aggregation. Incubation of 20 μM solutions of GLP-1RAs were performed as above in the absence or presence of 2 μM Aβ_42_. Once aggregation was completed in all samples (as assessed using tracer samples in adjacent wells), solutions were collected, centrifuged at 18,000*g* for 15 min, and supernatants carefully collected and measured using absorbance spectroscopy from 270 to 750 nm (Figure S18a). Spectra were highly similar when semaglutide, liraglutide, or tirzepatide were incubated with or without Aβ_42_, and A_280_ was largely unchanged by incubation with Aβ_42_ (Figure S18b). These results provide evidence that GLP-1RAs do not appreciably pellet with Aβ_42_ fibrils, suggesting that the inhibitory effects of GLP-1RAs arise from their transient interactions with Aβ_42_ species.

The combined inhibition of primary and secondary nucleation suggests that liraglutide is most likely interacting with an on-pathway aggregation intermediate formed by both nucleation processes, given this would require a single binding target rather than multiple to achieve the observed inhibitory effect, rather than its general binding to the fibril surface that would result in liraglutide entering the insoluble fraction. It is also possible that the inhibition of secondary nucleation by liraglutide is caused by the tight binding of this GLP-1RA to defect sites^38^ that occurs at very low stoichiometries below the limit of detection in these measurements. Lastly, as our data show that semaglutide and tirzepatide do not alter fibril structures (AFM, FTIR, and seeding capacity), it is likely that they do not incorporate into the fibril core structure. Liraglutide alters fibril morphology in a manner consistent with secondary nucleation inhibition, reduces seeding capacity even when added to preformed fibrils, and decreases fibril cross-sectional height and increases their lengths all without copelleting with fibrils. These observations support the situation in which liraglutide interacts with sites responsible for secondary nucleation, rather than becoming incorporated into the fibril core.

### Consideration of GLP-1RA-Induced Anti-Aggregation versus Competitive Aggregation

As GLP-1RAs can self-assemble,^39^ such species could result in the inhibition of Aβ_42_ aggregation via competitive aggregation. In the competitive aggregation model, GLP-1RAs self-assemble into micelles, oligomers, or fibrils that sequester Aβ_42_ monomers, reducing the effective concentration available to aggregate. In this scenario, aggregation slows not because the microscopic mechanisms of aggregation are specifically inhibited, but because Aβ sticks to GLP-1RA assemblies. In this case, kinetic effects could mimic inhibition without an underlying change in the microscopic rate constants due to a direct inhibition effect.

Several lines of evidence support GLP-1RAs herein exert direct anti-aggregation effects. Solubility tests for solutions of GLP-1RAs using absorbance spectroscopy showed minimal light scattering that would be consistent with an appreciable concentration of insoluble particles. Moreover, spectra were unchanged by centrifugation, which would remove fibrillar GLP-1RAs if present (Figure S7). This is further supported by seeding capacity control experiments (Figures S14 and S16). Subsequent tests found GLP-1RAs remain in the soluble fraction during the aggregation reaction and do not appreciably copellet with Aβ_42_ fibrils (Figure S18). For GLP-1RAs alone, ThT and Congo red fluorescence signal increases over time that may be indicative of oligomer or fibril formation were not seen (Figures S5d and S6), and GLP-1RA fibril formation was only observed as a very rare event by AFM for liraglutide (Figures 4 and S13). Final ThT plateau signals in the unseeded aggregation reactions were not decreased by semaglutide, tirzepatide, or liraglutide (Figure S2), which would be likely upon monomer sequestration as shown previously for a small molecule.^41^ Indeed, the kinetic signatures caused by GLP-1RAs are not easily explained by Aβ_42_ sequestration. We observed semaglutide and tirzepatide inhibited predominantly primary nucleation (Figure 2), whereas liraglutide targeted predominantly primary but also secondary nucleation (Figure 3), all with distinct IC_50_ values for different microscopic steps, whereas competitive aggregation would be expected to reduce effective monomer concentration and slow aggregation across all the seeded and unseeded assays. By DLS, semaglutide assembled into species that were larger than liraglutide in solution (Figures S11 and S12). Under a competitive aggregation model, semaglutide may be expected to be more potent, but it was ∼500,000× weaker than liraglutide at delaying primary nucleation. Lastly, Aβ_42_ fibrils coexist with a constant concentration of free monomeric and oligomeric Aβ_42_, and under standard in vitro conditions, fibril elongation and depolymerization can be considered in equilibrium. An absence of fibril disaggregation by GLP-1RAs (Figure S17) further suggests their assemblies are not functioning by sequestering free Aβ_42_, as was observed previously for claramine which stabilizes soluble protein species and reduces the free monomer concentration so that the depolymerization rate exceeds that of the elongation rate.^29^

### GLP-1­(7–37) Inhibits Aβ_42_ Aggregation by Perturbing Predominantly Elongation

GLP-1RAs have been carefully optimized to maintain partial or complete sequence homology with GLP-1(7–37), with modifications that enhance their biological activity and reduce their hydrolysis by dipeptidyl peptidase-4 (DPP-4), thereby extending their half-life.^21^ The half-life of GLP-1(7–37) is ∼2 min, exenatide is 2–4 h, liraglutide is ∼13 h, dulaglutide is ∼5 days, semaglutide is 7 days, and tirzepatide is 5 days.^42–44^ While our in vitro assays lack DPP-4, we nonetheless sought to determine the impact of human GLP-1(7–37) on Aβ_42_ aggregation. ThT kinetic experiments were performed exactly as with the above GLP-1RAs, using 0%, 5%, and 25% seeds and with 0 to 20 μM concentrations of GLP-1(7–37). GLP-1(7–37) exerted dose-dependent, large inhibitory effects in all unseeded and seeded assays (Figure S19). Analyses were conducted as before, and the unseeded data were best described by an impact on only elongation with *K*_IE_ = 0.771 μΜ (Figure S20). Finally, fitting the 5% seeded data, where *k*_*n*_ is not rate limiting, confirmed the best fits were obtained when perturbing only elongation, with *K*_IE_ = 0.520 μΜ (Figure S20). The data therefore support that GLP-1(7–37) inhibits Aβ_42_ aggregation mainly by suppressing elongation under these conditions.

## Discussion

Given the available preclinical and clinical reports supporting the potential relevance of GLP-1RAs in the treatment of AD, which were comprehensively reviewed in Dec. 2025 by Sabbagh et al.,^8^ our results provide fundamental insight into how these FDA-approved therapeutics for T2DM, cardiovascular disease, and weight loss can directly interact with Aβ_42_ and cause anti-aggregation effects. While exenatide and dulaglutide had minimal impact on Aβ_42_ aggregation under the conditions used herein, semaglutide, tirzepatide, and liraglutide all significantly delayed the self-assembly of this AD-linked peptide likely by targeting primary nucleation reactions in Aβ_42_ fibril formation (Figure 7a). The *K*_IP_ values obtained from the kinetic models were submicromolar for semaglutide and tirzepatide and subpicomolar for liraglutide. Only for liraglutide was a modest impact on secondary nucleation appreciated, with a supermicromolar *K*_IS_ value. These GLP-1RAs did not appreciably copellet with insoluble Aβ_42_ aggregates. Fibrils formed in the presence of semaglutide and tirzepatide were largely comparable to unmodified Aβ_42_ fibrils in terms of their maturity (cross-sectional heights), tortuosity, secondary structure, and templating efficiency. Fibrils formed in the presence of liraglutide were significantly less mature, with increased tortuosity and length relative to unmodified Aβ_42_ fibrils. Liraglutide also decreased the templating efficiency of Aβ_42_ fibrils (Figure 7b), through a mechanism consistent with the blocking of secondary nucleation sites, as supported by a range of biophysical assays.

GLP-1­(7–37) and liraglutide potently inhibited Aβ_42_ aggregation, while the effects of semaglutide and tirzepatide were less dramatic. Liraglutide shares 97% sequence homology with GLP-1(7–37), with one amino acid substitution of Lys34 to Arg34, and its ε-amine of Lys26 carries a palmitoyl fatty acid attached by γ-Glu (Figures 1 and S1). The addition of the 16-carbon saturated fatty acid increases the hydrophobicity of liraglutide relative to GLP-1(7–37), and enhanced amphiphilic interactions may play a role in its ability to interact with both soluble and insoluble Aβ_42_ to target predominantly primary nucleation with a modest impact on secondary nucleation, whereas GLP-1(7–37) inhibited predominantly elongation. Semaglutide also replaces Ala8 with α-aminoisobutyric acid (Aib), and its Lys26 carries a steric C18 fatty diacid connected via γ-Glu and two dioxaoctanoic acid linker moieties. Compared to semaglutide, tirzepatide features two Aib substitutions, additional amino acid changes, and a C20 fatty diacid. Both semaglutide and tirzepatide showed minimal ability to change the properties of Aβ_42_ fibrils, and they did not overtly influence seeded aggregation reactions.

While this study was limited to a subset of FDA-approved GLP-1RAs, we envision that future GLP-1RA derivatives can be rationally designed to systematically establish structure–activity relationships and optimize their anti-amyloid activity. Secondary nucleation is thought to be the step most responsible for Aβ_42_ oligomer generation and toxicity. Solely inhibiting primary nucleation is still expected to be beneficial since it delays oligomer formation and reduces toxicity, but doing so ultimately does not appreciably decrease the number of oligomers formed given enough time. Inhibiting elongation, however, may increase the number of oligomers formed and toxicity over time, as doing so redirects the reactive flux toward secondary nucleation.^26,45^ Future development of anti-amyloid GLP-1RAs would likely focus on optimizing the potency of secondary nucleation inhibition that was observed for liraglutide, since that step is most responsible for cytotoxic oligomer production. This has been shown previously for small-molecule inhibitors of Aβ_42_ aggregation.^25,26^ This could potentially be accomplished by changing the linkers and fatty acids attached to Lys26, or by mutating other residues in the GLP-1RA sequence.

It is possible that the less mature and longer fibrils formed in the presence of liraglutide could have differing abilities to induce cytotoxicity relative to unmodified fibrils. Moreover, our DLS studies showed that the incubation of liraglutide with Aβ_42_ oligomers resulted in a reduction in their observed size, implying that liraglutide directly interacts with oligomeric species. Also considering observations that toxic oligomers can detach from the ends of amyloid fibrils,^46^ detailed cytotoxicity studies are needed in the future to assess the biological consequences of the liraglutide-induced remodeling of fibrils and stabilized Aβ_42_ oligomers. While AD is primarily characterized by the accumulation of Aβ plaques and tau neurofibrillary tangles, common copathologies exist, such as α-synuclein in Lewy bodies. Similarly, Parkinson’s disease is primarily characterized by Lewy bodies of α-syn, but Aβ and tau copathologies are common.^47^ Semaglutide was found to decrease levels of aggregated α-synuclein and protect against neurodegeneration, among other effects, in the 6-OHDA Parkinson’s disease rat model.^48^ The extent to which GLP-1RAs can exert direct impacts on the aggregation of other amyloidogenic proteins is not well undersood, and this is relevant considering groups have proposed that GLP-1RAs could be useful to treat Parkinson’s disease through their systemic mechanisms of action.^8^

Breakdown of the BBB is linked to AD and chronic inflammation in the brain.^49,50^ Defense against neuroinflammation depends on the BBB.^8^ Liraglutide has been shown to preserve BBB integrity in a rat model of traumatic brain injury.^51^ BBB-penetration into key brain regions is important for GLP-1RAs to directly interact with Aβ_42_ in the human brain. Preclinical studies suggest that the GLP-1RAs tested here can traverse the BBB, but notable differences exist in the rates at which dulaglutide and tirzepatide cross.^8,52–56^ GLP-1(7–37) can also traverse the BBB.^52^ Exendin-4, the naturally occurring peptide from which exenatide is based, crosses the BBB, and it has been suggested that treatment can improve BBB integrity in diabetic rats.^57,58^ GLP-1RA uptake in the mouse brain requires GLP-1R and is limited to circumventricular areas and regions near blood vessels, which may leverage murine tanycytes, specialized ependymal cells that support BBB integrity. Liraglutide was shuttled in that study to the hypothalamus by these specialized tanycytes, bypassing the BBB.^59^ Silencing *Glp1r* expression reduced liraglutide transport into the mouse brain and suppressed the downstream effects of liraglutide on mouse metabolism.^8,59^

**Figure 7:**
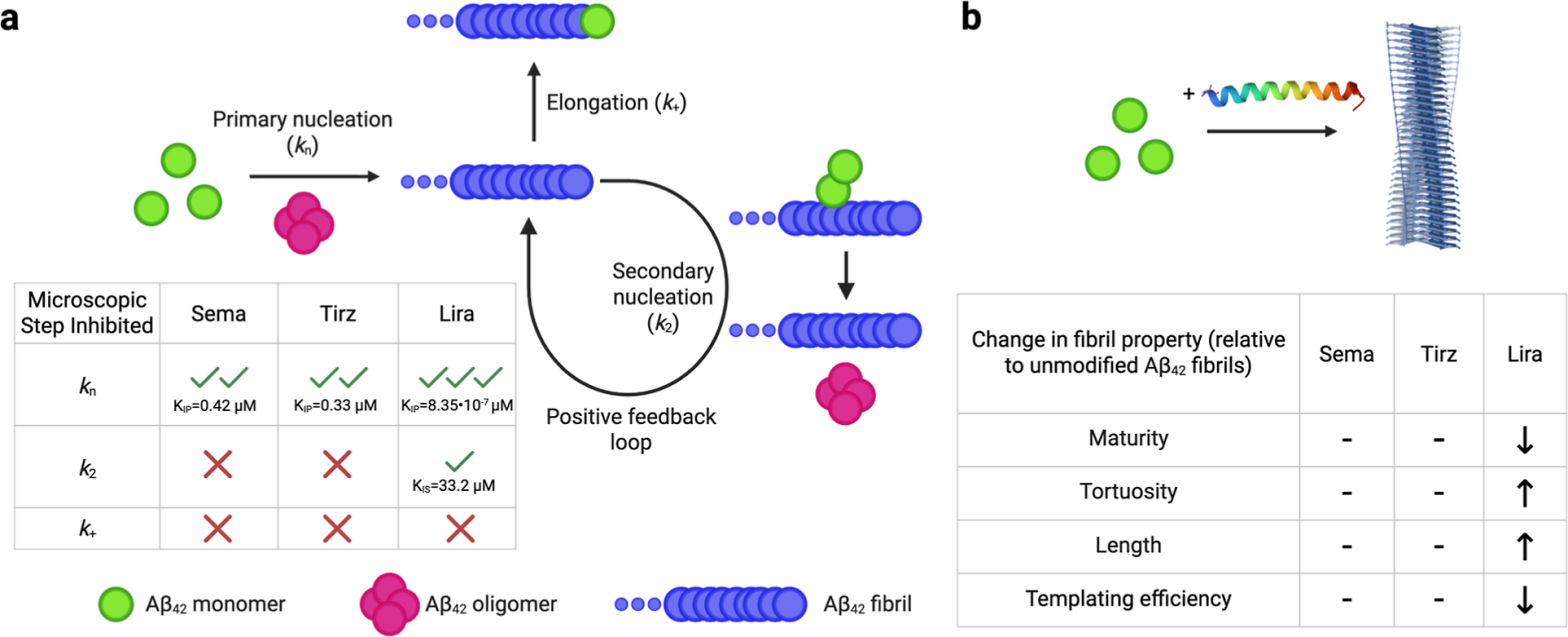
Summary of the impact of semaglutide, tirzepatide, and liraglutide on Aβ_42_ aggregation. (a) Semaglutide, tirzepatide, and liraglutide inhibit Aβ_42_ aggregation with differing potencies by predominantly targeting primary nucleation reactions. Liraglutide also modestly inhibits secondary nucleation reactions. (b) Aβ_42_ fibrils formed in the presence of liraglutide are less mature, more tortuous, longer, and have a lower ability to passively self-replicate. Arrows indicate statistically significant changes in the indicated parameter (see Results). Dashes indicate that no statistically significant change was observed. Semaglutide structure is shown from PDB: 7KI0_5. Created with BioRender.com.

In mice, VivoTag-S 750 liraglutide (liraglutide^750^) accessed all circumventricular organs, including the zona interna of the median eminence, the subfornical organ, the organum vasculosum of the lamina terminalis, the choroid plexus, and it was abundant in hypothalamic regions protected by the BBB, including the arcuate nucleus, the paraventricular nucleus, and the supraoptic nucleus and supraoptic decussation. These signals were lost in *Glp1r* ^–/–^ mice. Comparable hypothalamic distributions were also observed in rats.^19^ In rats, LC–MS/MS analyses determined liraglutide achieved plasma concentrations of 102,801.74 ± 6627.17 ng/mL and 181,525.94 ± 8838.02 ng/mL after 5 mg/kg intravenous and 500 mg/kg subcutaneous injections, respectively, with a low brain tissue-to-plasma partition coefficient below 0.00031. Despite this, liraglutide was detected and different in various regions of the brain, with the highest levels found in the hypothalamus, followed by the cerebellum, and cerebrum.^60^

The absence of a detailed characterization of the BBB permeability of GLP-1RAs in humans, alongside the potentially short half-life of these peptides in the brain, are major limitations to understanding the potential biological and therapeutic relevance of this class of therapeutics in the setting of AD. Once weekly semaglutide at 1.0 mg achieved a steady state plasma concentration of 47.4 nM in healthy Chinese adults.^61^ Some evidence suggests semaglutide enters the human brain, but only reaches 0.4% of plasma levels after three months of treatment.^62^ With multiple dose administration of 15 mg of tirzepatide, mean steady state peak plasma concentration was 1990 ng/mL, while average systemic exposure was 1480 ng/mL.^63^ Direct evidence for tirzepatide accumulation in the human brain is limited. Following 1.8 mg and 3.0 mg doses of liraglutide to obese patients, maximum plasma concentrations were 30.2 and 39.2 nM, respectively.^64^ In one study, eight T2DM patients were treated with 1.8 mg liraglutide for 14 months. Mean plasma-liraglutide was 31 nM, and mean CSF-liraglutide was 6.5 pM.^65^ The latter would also likely increase with the 3.0 mg dose.

These animal studies and clinical trials imply it is possible that liraglutide can accumulate in the brain at concentrations near our kinetically quantified *K*_IP_, suggesting liraglutide may reach amounts in the brain sufficient to inhibit Aβ_42_ primary nucleation. We emphasize, however, the exact concentration of each GLP-1RA in the brain as it relates to the various human dosing regimens remains poorly understood. It will likely be necessary to optimize GLP-1RA potency for *K*_IS_, as well as BBB permeability, to see a major effect on secondary nucleation. The former is achievable using rational drug discovery approaches,^25,26^ and the latter could be promoted by modifying liraglutide to reduce its size, charge, acyl chain length or branching to target its hydrophobicity, or through modern peptide brain delivery methods, for example emerging liposome-based strategies to overcome BBB transport deficiencies.^66^ Future work to determine the parts of liraglutide critical for the inhibition of secondary nucleation may facilitate its reduction in size or hydrophobicity, which could promote its ability to traverse the BBB or enter through different mechanisms.

The potential effects of GLP-1RAs on humans are diverse. Compared to usual care, GLP-1RAs use in diabetic patients was associated with reduced risk of substance use and psychotic disorders, seizures, coagulation disorders, cardiometabolic disorders, infectious illnesses, several respiratory conditions, and neurocognitive disorders, including AD. An increased risk of gastrointestinal disorders, hypotension, syncope, arthritic disorders, nephrolithiasis, interstitial nephritis and drug-induced pancreatitis was also associated with GLP-1RA use.^67^ Preclinical studies show that GLP-1RAs attenuate neuroinflammation by targeting neurons, astrocytes, and microglia.^8,68^ The benefits of GLP-1RAs to the cardiovascular system may reduce both large and small vessel disease, which contribute to vascular cognitive impairment and AD.^8,69^ AD pathogenesis includes the following modern hypotheses: cholinergic, glutamate excitotoxicity, amyloid, tau, inflammatory, microbiota-gut-brain axis, abnormal autophagy, metal ion, and oxidative stress.^70^ As AD is the result of a complicated imbalance in multiple, interconnected systems that change over the course of the disease, a multipronged treatment regimen is likely necessary to comprehensively treat AD. While GLP-1RAs hold promise for delaying the onset of neurodegenerative diseases and influencing several pathways that are systemically dysregulated in AD, significant questions remain about their safety, efficacy, and optimal dosing.

The recent evoke and evoke+ phase III clinical studies of 14 mg daily oral semaglutide given for 104 weeks to patients with biomarker-confirmed MCI or mild AD dementia failed to slow the progression of AD, despite lower levels of biomarkers indicative of disease activity.^8^ As dose–responses are not established in humans for the neuroprotective actions of GLP-1RAs, the trials may have failed to benefit AD patients for several reasons, including suboptimal circulating levels of oral semaglutide and insufficient penetration of semaglutide into key brain regions.^8^ While the “evaluating liraglutide in Alzheimer’s disease” (ELAD) phase 2b study of daily injected liraglutide administered for 52 weeks to mild to moderate AD patients showed no significant changes in cerebral glucose metabolism, slowed cognitive deterioration and brain volume loss were observed compared to placebo. Based on the timing of patient improvement, it was suggested that GLP-1RAs may positively influence congition in AD over an extended time of treatment.^14^ Treatment was considered safe and well-tolerated in these trials.^14,71^ Herein, liraglutide inhibited primary nucleation with markedly greater potency in comparison to semaglutide. Moreover, only liraglutide inhibited the ability of fibrils to passively self-replicate. In fact, its ability to attenuate fibril seeding capacity was strongest when it was added to unmodified preformed Aβ_42_ fibrils. The striking potency difference for *K*_IP_ between semaglutide and liraglutide, combined with the potential dual mechanism of action of liraglutide, may help explain in some part these differences in clinical outcomes. It remains to be seen if liraglutide reduces amyloid burden in AD patients through inhibitory mechanisms related to its potential ability to improve cognitive outcomes. Nonetheless, a recent meta-analysis of randomized clinical trials found treatment with GLP-1RAs was linked with a significant reduction in dementia or cognitive impairment,^72^ which supports the potential relevance of the reported mechanistic insights. In the above considerations, we reiterate BBB penetration differences between different GLP-1RAs are a potential confounding factor when comparing in vitro potency to clinical efficacy.

Collectively, our findings may highlight important advantages of liraglutide over the other tested GLP-1RAs. Initial promise in preclinical pipelines and from retrospective studies, as well as emerging clinical trial results, call for further insights into the mechanistic underpinnings of GLP-1RAs in the context of AD in these complicated biological systems. Side effects of GLP-1RAs at optimized therapeutic doses must also be considered, such as unintended weight loss, ocular changes, nausea, headaches, and others.^73,74^

## Conclusion

In addition to the previously postulated protective mechanisms of GLP-1RAs against AD that are being explored in clinical trials, the work presented herein provides direct evidence of the ability of GLP-1RAs to inhibit Aβ_42_ aggregation. Our results show select current-generation GLP-1RAs inhibit Aβ_42_ aggregation by attenuating predominantly primary nucleation reactions, which may contribute to their hypothesized ability to protect against AD if they are administered sufficiently early in the course of disease. In addition to its overtly enhanced potency to inhibit primary nucleation compared to semaglutide or tirzepatide, liraglutide can uniquely also inhibit secondary nucleation reactions and dampen the intrinsic ability of Aβ_42_ fibrils to passively self-replicate, including when introduced after Aβ_42_ fibrils have formed. Future work will continue to address the impact of these highly relevant therapeutics in the setting of AD, as well as other neurodegenerative diseases like Parkinson’s disease.^8^

## Experimental Materials and Methods

### Reagents

GLP-1RAs were acquired from Sigma-Aldrich (MO, USA; semaglutide acetate, 99.45%, TA9H98DB1905; tirzepatide acetate, 98.05%, TA9H97F3118A; liraglutide, ≥95%, SML3925; exenatide acetate, 98%, AABH9A21950A; dulaglutide, 95%, ATEH9A573F59). Human GLP-1 recombinant protein, PeproTech (130-08-1MG, ≥98%) was purchased from Thermo Fisher Scientific (MA, USA). Aliquots were prepared at a concentration of 2 mM in 100% dimethyl sulfoxide (DMSO) and stored at −20 °C. Freeze/thaw cycles were avoided. Samples containing proteins were prepared or stored in Eppendorf LoBind Tubes (Hamburg, Germany).

### Protein Preparation

One mL of 6 M guanidine hydrochloride was added to 1 mg of >97.0% pure Aβ_42_ lyophilized peptide (Sigma-Aldrich, ultrapure recombinant human peptide, AG968, molecular weight of 4514 g/mol), gently ultrasonicated in a water bath, and this solution was equilibrated on ice for 1 h before further purification. Next, size exclusion chromatography was carried out using an AKTA pure chromatography system (Cytiva, MA, USA) in 20 mM sodium phosphate buffer, 200 μM EDTA, pH 8.0, using a Superdex 75 Increase 10/300 column at a flow rate of 0.5 mL/min. EDTA was used to chelate any residual metal ions in the buffer, which are known to affect Aβ aggregation.^30,75^ The peak containing the monomeric peptide was collected and immediately diluted to 20 μM in the same cold phosphate buffer and kept on ice.

### Aggregation Assays Using ThT

Samples of monomeric Aβ_42_ were prepared on ice in Protein LoBind Eppendorf tubes with the appropriate amounts of each GLP-1RA and/or preformed fibrils. ThT was added from a 2 mM stock to give a final concentration of 20 μM. All samples were prepared at constant 1% (v/v) DMSO, which has been shown not to impact the aggregation kinetics of Aβ_42_,^26^ unless otherwise stated. Care was taken to avoid the production of bubbles at all stages of sample preparation. Samples were then transferred to a 96-well half area, low-binding, clear-bottom, PEG-coated plate at a final volume of 100 μL per well (Corning 3881). ThT fluorescence was monitored in triplicate per sample as measured using bottom optics in a plate reader (CLARIOstar Plus, FLUOstar Omega, or VANTAstar from BMG Labtech, Ortenberg, Germany) with 440 and 480 nm excitation and emission filters, respectively.^26^ Aggregation was initiated by transferring the 96-well plate to a plate reader at 37 °C. Only quiescent aggregation (e.g., no shaking in the plate reader) was followed in all experiments.

For the seeded experiments, freshly prepared fibrils were generated as above immediately before each experiment. ThT fluorescence was monitored over time to confirm that fibrils formed. As needed, identical samples in adjacent wells were aggregated into fibrils without ThT for use in label-free studies. The samples were then collected from the wells into low-binding tubes unless otherwise stated. The effects of the GLP-1RA in unseeded and seeded assays were confirmed in at least three independent experiments.

### Aβ_42_ Kinetic Analyses

The time evolution of the total fibril mass concentration, M­(t), is described by the following integrated rate law^34^

$$\frac{M\left(t\right)}{M\left(\infty \right)}=1-{\left(\frac{B_{+}+C_{+}}{B_{+}+C_{+}e^{kt}}\frac{B_{-}+C_{+}e^{kt}}{B_{-}+C_{+}}\right)}^{k_{\infty }^{2}/\kappa {\mathrm{k̃}}_{\infty }}e^{-k_{\infty }t}$$

where the kinetic parameters *B*_±_, *C*_±_, κ, *k*_∞_ and ${\mathrm{k̃}}_{\infty }$ are functions of the two combinations of the microscopic rate constants *k*_+_ *k*_2_ and *k*_+_ *k*_*n*_, where *k*_*n*_, *k*_+_, and *k*_2_ are the primary nucleation, elongation and secondary nucleation rate constants, respectively (see ref 34 for explicit expressions). The perturbations induced by the GLP-1RAs were resolved by fitting experimental data to the above equation to identify which rate parameters are affected by the compound. The fits of kinetic data without and with compounds were performed using the AmyloFit platform,^27^ which uses a basin-hopping algorithm to find the best fit to the data using the above equation. Different combinations of altered rate parameters can be investigated using this approach (e.g., the inhibitor only affects *k*_*n*_ according to a rapid binding equilibrium model with IC_50_, while keeping all other parameters fixed for a compound inhibiting primary nucleation only).

### Aggregation Assays Using Congo Red

Samples of monomeric Aβ_42_ were prepared as above. Congo red was added from a 2 mM stock to give a final concentration of 20 μM. All samples were prepared at constant 1.1% (v/v) DMSO. Congo red fluorescence was monitored in triplicate per sample as measured using bottom optics in a plate reader (CLARIOstar Plus or FLUOstar Omega from BMG Labtech, Ortenberg, Germany) with 544 and 590 nm excitation and emission filters, respectively.

### Absorbance Spectroscopy

Solutions of GLP-1RAs alone were prepared at 20 μM in the above buffer and measured using a Jasco V-630 Spectrophotometer and a quartz cuvette. Spectra were acquired from 270 to 750 nm to assess the solubility of the GLP-1RAs. Samples were centrifuged at 18,000*g* for 15 min and spectra recorded again to check for changes in UV–vis absorbance indicative of the presence of sedimented insoluble particles, which were not observed. All spectra were baseline corrected at 330 nm, as shown. A 1% DMSO solution was used as the blank.

In a separate set of experiments, GLP-1RAs were incubated as above in the absence or presence of Aβ_42_. All solutions were collected once samples containing Aβ_42_ had entered the plateau phase of aggregation (according to tracer samples), centrifuged at 18,000*g* for 15 min, and supernatants were carefully collected for analysis using a Jasco V-630 Spectrophotometer and a quartz cuvette. Spectra were acquired from 270 to 750 nm to assess if GLP-1RAs appreciably pellet with Aβ_42_ fibrils. All spectra were baseline corrected at 330 nm, as shown. We confirmed that the supernatant of a control sample containing only 2 μM Aβ_42_ (in 1% DMSO) had a negligible A_280_ signal since fibrillar aggregates were pelleted out, and this sample was used as the blank.

### Atomic Force Microscopy (AFM)

Solutions containing aggregated Aβ_42_ were deposited on mica in the absence of ThT once all samples had entered the plateau phase of aggregation. The mica substrate was positively functionalized prior to sample deposition by the incubation of a 20 μL drop of 0.5% (v/v) APTES in ultrafiltered Elga water (Purelab Classic, CLXXXUFM2-US, Elga LabWater, IL, USA) for 2 min at ambient temperature, rinsed twice with water, and then dried by the passage of a gentle flow of gaseous nitrogen.^76^ AFM samples were prepared on the freshly functionalized mica surfaces by the deposition of a 30 μL drop of protein (2 μM Aβ_42_, in monomer equivalents) for 5 min. Salts were washed away with water twice, the surface was dried with gaseous nitrogen, and the samples were stored in sealed containers until imaging was carried out using a Park NX10 Atomic Force Microscope (Park Systems, Suwon, South Korea) operating in noncontact mode with scan rates <0.5 Hz and PPP-NCHR cantilevers (<10 nm tip radius, 42 N/m, 330 kHz from Park Systems, as manufactured by Nanosensors, Neuchatel, Switzerland). Care was taken to ensure the phase change upon imaging was less than Δ20° during the interaction of the tip with the sample,^30^ which enabled us to consistently compare the morphology of the different samples.

AFM height maps (8 bit TIFFs) were processed using a custom MATLAB code to determine fibril tortuosity and length. The two end points for each fibril were manually selected on the image. The normalized height map (Znorm) was thresholded to isolate elevated fibrillar material, cleaned to remove small noise objects, and skeletonized (bwskel) to obtain a one-pixel-wide “backbone”. The height-weighted backbone shortest path prioritized high-intensity (taller) pixels. The resulting backbone coordinates (*x*_*i*_ *,y*_*i*_) were used to calculate the total path length as the sum of segment distances. Fibril tortuosity was defined as the ratio of backbone path length to direct length (Euclidean distance between end points). Representative images and additional details are provided in Figures S21 and S22. Fibril cross-sectional heights were determined in XEI, an Image Processing Program for SPM data from Park Systems. Where different cross-sectional heights were observed within one fibril, each height was recorded once. For all analyses above, regions of fibrils crossing over one another were not considered. Sample sizes were 212, 98, 214, and 69 fibrils for Aβ_42_, Aβ_42_ + Sema, Aβ_42_ + Tirz, and Aβ_42_ + Lira, respectively, in the tortuosity and length analyses. Following the procedure described above for cross-sectional height, 95, 173, 138, and 114 fibril cross sections were analyzed for Aβ_42_, Aβ_42_ + Sema, Aβ_42_ + Tirz, and Aβ_42_ + Lira, respectively.

### Fourier Transform Infrared Spectroscopy (FTIR)

Aβ_42_ was aggregated at a concentration of 2 μM in the absence or presence of a 1:10 molar ratio of Aβ_42_ to GLP-1RA until all samples had finished aggregating, as confirmed using samples prepared in parallel with ThT. Aggregation mixtures were collected from 96-well plates (Corning 3881), and solutions were centrifuged for 15 min at 18,000*g* (20 °C). Supernatant was carefully removed, each pellet was resuspended in 10 μL pure water such that the final concentration of Aβ_42_ was ∼400 μM, and 2 μL spots were deposited and allowed to dry on a Nicolet iS20 FTIR (Thermo Fisher Scientific, Waltham, MA, USA). Spectra were recorded with a resolution of 4 cm^–1^ and 512 scans and were smoothed (19 points, 9.160 cm^−1^) prior to taking the second derivative using a second order, 13-point Savitzky–Golay filter in integrated OMNIC spectra software. Data shown are representative of two independent experiments.

### Preparation of Stabilized Oligomers

Lyophilized Aβ_42_ (Sigma-Aldrich, ultrapure recombinant human peptide, AG968) was dissolved in 100% hexafluoro-2-isopropanol (HFIP) to a concentration of 1.0 mM, and the solvent was then evaporated. Aβ-derived diffusible ligands (ADDLs) were prepared from Aβ_42_ according to Lambert’s protocol.^30,77^

### Dynamic Light Scattering

Oligomers were formed and incubated with GLP-1RAs for 1 h at 37 °C. A 4-fold molar excess of each GLP-1RA to Aβ_42_ was not exceeded to keep the DMSO at, and not above, a constant concentration of 2% v/v. DLS measurements were performed with automatic parameters for attenuator and cell position at 25 °C using the Malvern Zetasizer Nano S instrument (Malvern, Worcestershire, UK) equipped with a Peltier temperature controller. A low-volume (70 μL) disposable cuvette was used (BRAND, Wertheim, Germany). Measurements were also carried out for semaglutide, tirzepatide, and liraglutide at 37 °C.

### Disaggregation Assays

Using the same buffer as before, fibrils were formed at a concentration of 2 μM and subsequently combined with 20 μM ThT in the absence or presence of 20 μM concentrations of semaglutide, tirzepatide, or liraglutide. As before, the ThT signal was monitored over time at 37 °C with 440 and 480 nm excitation and emission filters.

### Seeding Capacity Experiments

Label-free fibrils were formed at a concentration of 2 μM in the absence or presence of 2 μM or 20 μM concentrations of semaglutide, tirzepatide, or liraglutide in a 96-well plate (Corning 3881) using the same aggregation conditions as above. Preformed fibrils were also exposed to 2 μM or 20 μM concentrations of semaglutide, tirzepatide, or liraglutide under the same conditions. When all samples had entered the plateau phase of aggregation (as assessed by tracer samples in adjacent wells containing ThT, after ∼6 h), solutions were collected and centrifuged at 18,000*g* for 15 min. Supernatants were discarded, and pellets containing insoluble aggregates were resuspended in the above phosphate buffer. The various stocks were then used to create solutions containing 2 μM fresh monomeric Aβ_42_ and 2%, 5%, or 10% (in monomer equivalents) of each fibril seed, and aggregation was followed using the same conditions as in the above kinetic assays. Negative control samples containing 20 μM concentrations of semaglutide, tirzepatide, or liraglutide in the absence of Aβ_42_ were treated exactly as the other samples and caused a negligible effect on Aβ_42_ aggregation.

### Statistics and Reproducibility

Data were analyzed in GraphPad Prism 10.6 (CA, USA) by one-way ANOVA followed by Dunnett’s post hoc comparison test, except where otherwise indicated, and *P* < 0.05 was accepted as statistically significant. Except where otherwise stated: kinetic experiments were performed with three technical replicates, and unseeded and seeded ThT kinetic experiments with semaglutide, tirzepatide, and liraglutide are representative of three independent experiments (defined herein as different protein purifications); Congo red assays were performed with three technical replicates and are representative of two independent experiments; AFM samples were prepared once at the end of the aggregation reaction and measured with the samples sizes listed above; UV–vis and FTIR experiments measured single spectra, and reproducibility was checked in two independent experiments; unique effects in DLS experiments were consistent in two independent experiments; seeding capacity experiments and associated controls were performed with three technical replicates and were confirmed in two independent experiments; GLP-1(7–37) ThT aggregation experiments were performed with three technical replicates and are representative of two independent experiments.

## Supplementary Material



## Data Availability

Data used in the manuscript are available from the corresponding author upon request.

## Notes

Supplementary Figures. S1: GLP-1RA sequences and side chain structures; S2: ThT assays for unseeded and seeded Aβ_42_ aggregation with semaglutide, tirzepatide, and liraglutide; S3: Exenatide and dulaglutide do not impact Aβ_42_ aggregation; S4: Half-times from Figures 2 and 3; S5: Congo red assays for unseeded Aβ_42_ aggregation with semaglutide, tirzepatide, and liraglutide; S6: Lack of detectable aggregation for semaglutide, tirzepatide, liraglutide, dulagultide, and exenatide alone in the ThT assays; S7: GLP-1RA solubility checks using absorbance spectroscopy; S8: Determination of global fitting constants for Aβ_42_ aggregation; S9: Kinetic fitting of the Aβ_42_ aggregation traces in the presence of semaglutide and tirzepatide indicates a decrease in *k*_n_, but not *k*_2_ or *k*_+_; S10: Seeding experiments confirm liraglutide has an impact on fibril-dependent microscopic processes in Aβ_42_ aggregation; S11: Dynamic light scattering measurements of stabilized Aβ_42_ oligomers exposed to GLP-1RAs; S12: DLS of GLP-1RAs at 37 °C; S13: Presence of rare fibrils in AFM images for liraglutide alone; S14: Control experiments for seeding capacity assays; S15: Liraglutide alone reduces the seeding capacity of Aβ_42_ fibrils; S16: Seeding capacity control experiments; S17: Semaglutide, tirzepatide, and liraglutide do not disaggregate Aβ_42_ fibrils; S18: GLP-1RAs remain largely soluble during Aβ_42_ aggregation; S19: ThT assays for unseeded and seeded Aβ_42_ aggregation with GLP-1(7–37); S20: GLP-1(7–37) inhibits Aβ_42_ aggregation by suppressing elongation; S21: Representative AFM images showing fibril analyses; S22: Fibril path length analysis (PDF)
