# Supplementary material for: Glucagon-Like Peptide‑1 Receptor Agonists Inhibit the Initiation of Toxic Amyloid-β42 Aggregation
Source: J Am Chem Soc. 2026 May 14;148(20):20582–99. doi: 10.1021/jacs.6c01289 (PMC13220265; doi:10.1021/jacs.6c01289)
Supplement: Supplementary file 1 [file ja6c01289_si_001.pdf]

Supplementary Information for:

Glucagon-like peptide-1 receptor agonists inhibit the initiation of toxic amyloid-β42 aggregation

Lucas B. Fallot[1,2,+], Carol A. Anderson[1,2,+], Johnathan R. Pinc[1,2,+], Alisdair Stevenson[3,4,+], Mary Claire Schleck[1,2], Ethan Hawryschuk[1,2], Owen Z. Li[1,2], Julia C. Palchak[1,2], Justin R. Toole[1], Robert W. Kubiak II[1], Alexander J. Dear[3,4], Thomas C. T. Michaels[3,4], Ryan Limbocker[1,2,*]

[1]Department of Chemical and Biological Science and Engineering, United States Military Academy, West Point, NY 10996, United States of America

[2]Photonics Research Center, United States Military Academy, West Point, NY 10996, United States of America

[3]Department of Biology, Institute of Biochemistry, ETH Zurich, Otto Stern Weg 3, 8093 Zurich, Switzerland

[4]Bringing Materials to Life Initiative, ETH Zurich, Otto Stern Weg 3, 8093 Zurich, Switzerland

[+]These authors contributed equally.

[*]Correspondence to: ryan.limbocker@westpoint.edu (R.L.)

a

	Number of Amino Acids	Sequence
**GLP-1 Analog Sequence**	31	HGEGTFTSDVSSYLEEQAAKEFIAWLVKGGG
**Linker/Junction Region**	16	GGGGSGGGGSGGGGSA
**Fc Hinge**	12	ESKYGPPCPPCP (disulfide bonds between cysteine residues 55 and 58)
**Fc Constant Heavy Chain 2 Domain**	89	APEAAGGPSVFLFPPKPKDTLMISRTPEVTCVVVDVSQEDPEVQFNWYVDGV EVHNAKTKPREEQFNSTYRVVSVLTVLHQDWLNGKEY
**Fc Constant Heavy Chain 3 Domain**	128	KCKVSNKGLPSSIEKTISKAKGQPREPQVYTLPPSQEEMTKNQVSLTCLVKGF YPSDIAVEWESNGQPENNYKTTPPVLDSDGSFFLYSRLTVDKSRWQEGNVFS CSVMHEALHNHYTQKSLSLSLG
**Total Monomer Amino Acids**	276	
**Total Dimer Amino acids**	552	

b

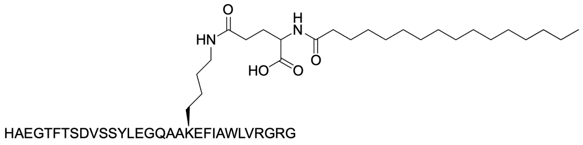

c

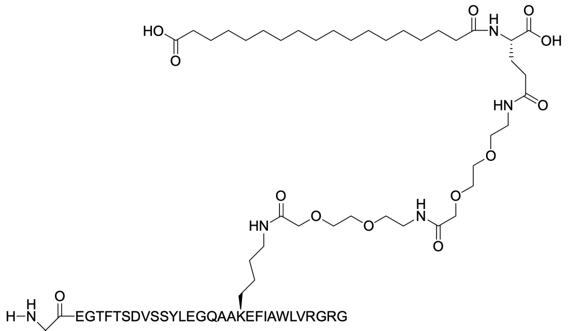

d

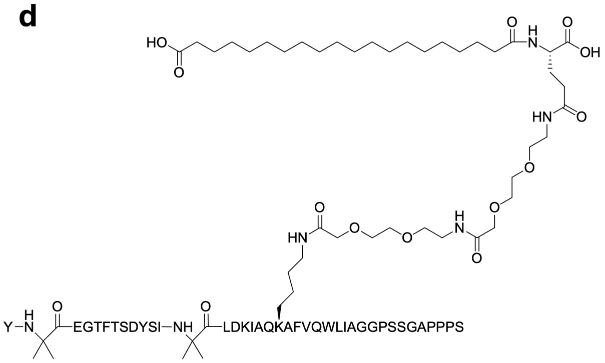

Figure S1: GLP-1RA sequences and side chain structures. Amino acid sequences and side chain structures for dulaglutide (**a**) (from patent US20230088005A1), liraglutide (**b**) (from patent WO2018104922A1), semaglutide (**c**) (from patent EP3398960B1), and tirzepatide (**d**) (from patent US9474780).

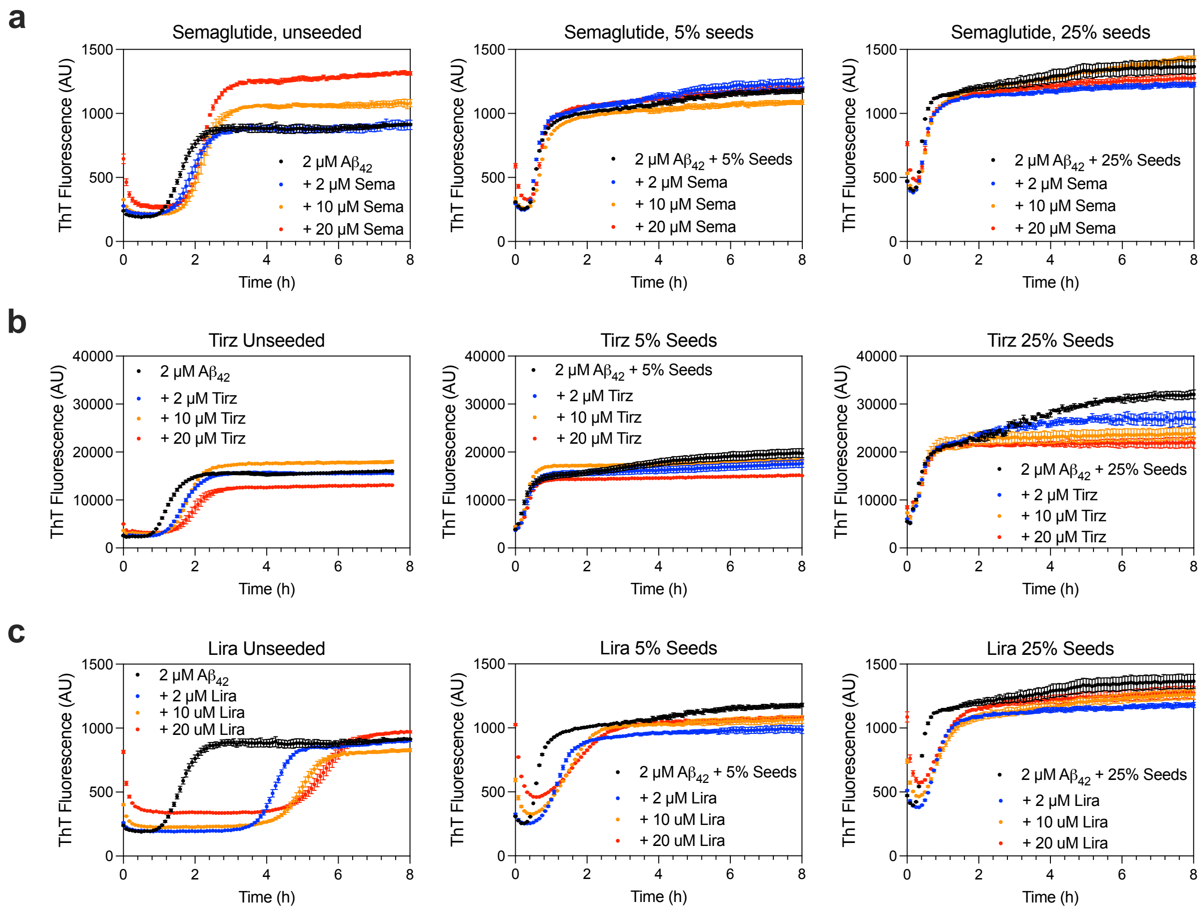

Figure S2: ThT assays for unseeded and seeded $A\beta_{42}$ aggregation with semaglutide, tirzepatide, and liraglutide. Experiments were performed using 2 μM $A\beta_{42}$ (in 20 mM sodium phosphate, 0.2 mM EDTA, 1% v/v DMSO, pH 8.0, quiescent) in the absence (black) or presence of 2 (blue), 10 (orange), or 20 μM (red) concentrations of semaglutide (Sema, **a**), tirzepatide (Tirz, **b**), or liraglutide (Lira, **c**) with no fibril seeds (left panels), 5% fibril seeds added at the start of the aggregation reaction (middle panels), or 25% fibril seeds added at the start of the aggregation reaction (right panels). Normalized unseeded data are shown and modeled in **Figures 2** and **3**. Error bars indicate s.e.m. of three technical replicates. Data are representative of at least three independent experiments.

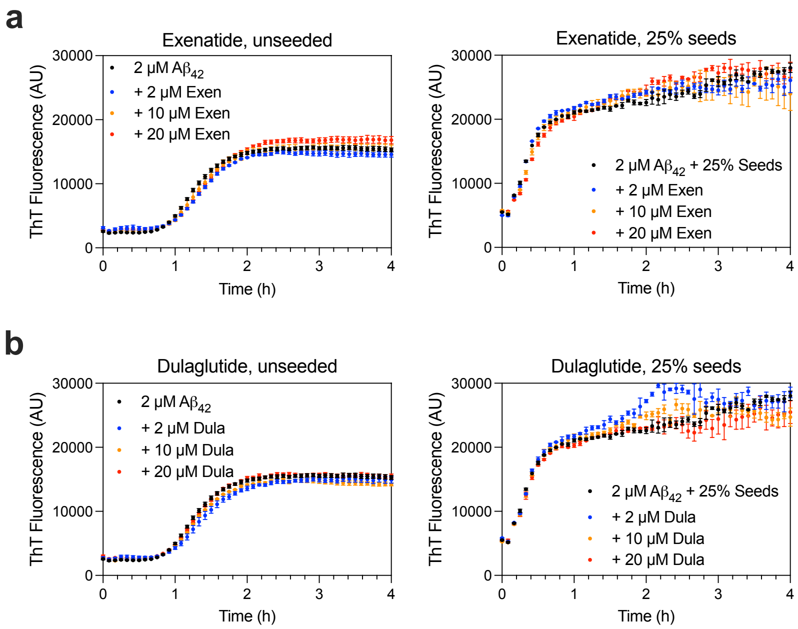

Figure S3: Exenatide and dulaglutide do not impact Aβ_{42} aggregation. Experiments were performed using 2 μM Aβ_{42} (in 20 mM sodium phosphate, 0.2 mM EDTA, 1% v/v DMSO, pH 8.0, quiescent) in the absence (black) or presence of 2 (blue), 10 (orange), or 20 μM (red) concentrations of exenatide (Exen) (**a**) or dulaglutide (Dula) (**b**) with no fibril seeds (left panels) or 25% fibril seeds added at the start of the aggregation reaction (right panels). Error bars indicate s.e.m. of three technical replicates.

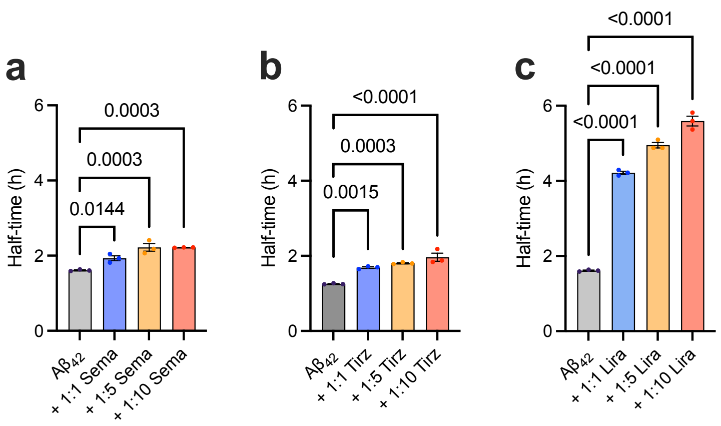

Figure S4: Half-times from Figures 2 and 3. Half-times calculated using AmyloFit (Meisl et al., *Nat. Protoc.*, 2016) for 2 μM $A\beta_{42}$ aggregating in the absence or presence of semaglutide (**a**), tirzepatide (**b**), or liraglutide (**c**) at 1:1 to 1:10 molar ratios of $A\beta_{42}$ to GLP-1RA. Error bars indicate s.e.m. of three replicates. Data were analyzed by one-way ANOVA with Dunnet's post-comparison test relative to $A\beta_{42}$ alone.

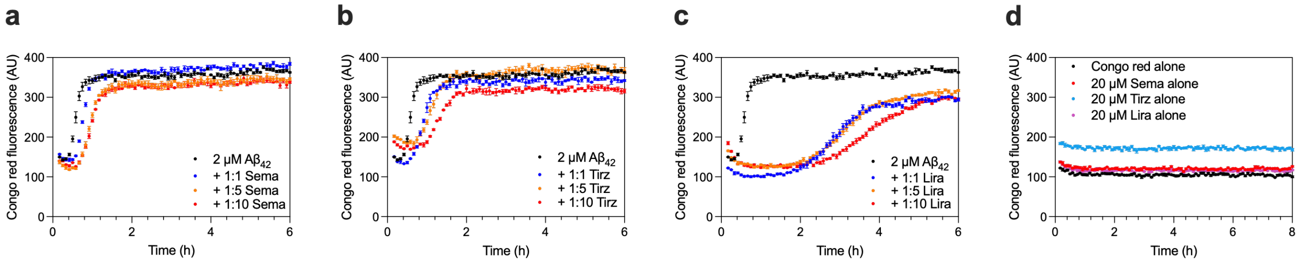

Figure S5: Congo red assays for unseeded Aβ_{42} aggregation with semaglutide, tirzepatide, and liraglutide. Experiments were performed using 2 μM Aβ_{42} (in 20 mM sodium phosphate, 0.2 mM EDTA, 1.1% v/v DMSO, pH 8.0, quiescent) in the absence (black) or presence of 2 (blue), 10 (orange), or 20 μM (red) concentrations of semaglutide (**a**), tirzepatide (**b**), or liraglutide (**c**). (**d**) No aggregation was detected by Congo red for 20 μM of each of these GLP-1RAs in the absence of Aβ_{42}. Data are representative of two independent experiments.

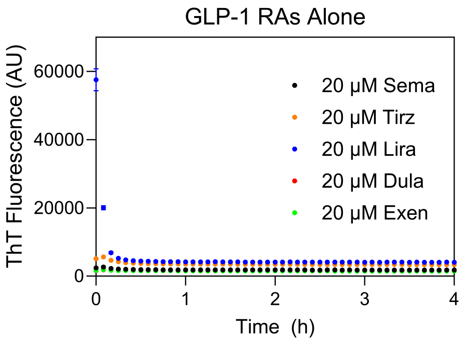

Figure S6: Lack of detectable aggregation for semaglutide, tirzepatide, liraglutide, dulaglutide, and exenatide alone in the ThT assays. Aggregation over time was not detected by ThT under the tested conditions for 20 μM of each of these GLP-1RAs in the absence of Aβ_{42}.

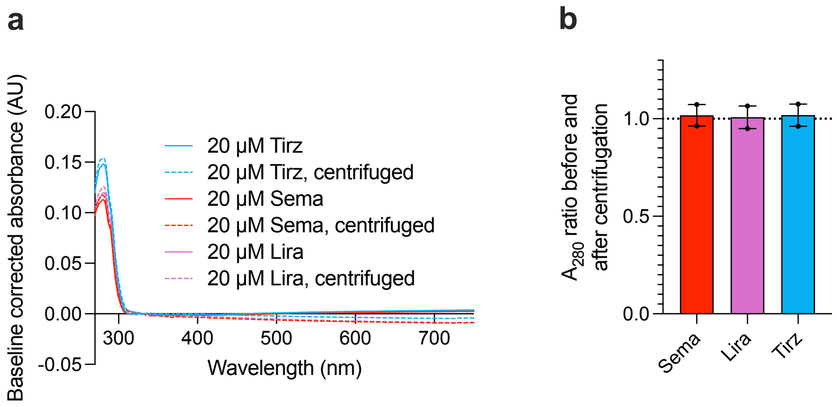

Figure S7: GLP-1RA solubility checks using absorbance spectroscopy. (**a**) GLP-1RA solutions at 20 μM (1% DMSO, room temperature) were measured from 270 to 750 nm using absorbance spectroscopy. Sharp peaks were observed with absorbance maxima near 280 nm (A_{280}) caused by GLP-1RAs. Spectra were baseline corrected at 330 nm, as shown. (b) A_{280} before and after centrifugation. Error bars indicate s.e.m. of two independent experiments.

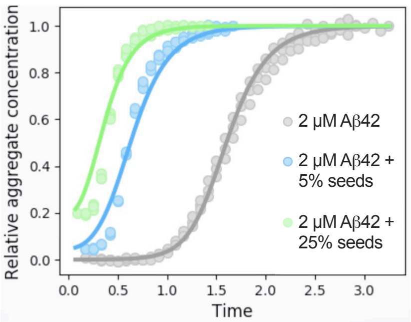

Figure S8: Determination of global fitting constants for Aβ_{42} aggregation. 2 µM Aβ_{42} (in 20 mM sodium phosphate, 0.2 mM EDTA, 1% v/v DMSO, pH 8.0, quiescent) was aggregated in the absence (grey) or presence of 5% (blue) or 25% (green) fibril seeds. These data were globally fit to determine the unified rate constants for Aβ_{42} aggregation under the conditions tested in the kinetic assays herein, yielding $k_n = 1.15 \cdot 10^4$ M$^{-1} \cdot$hr^{-1}, $k_2 = 5.99 \cdot 10^{13}$ M$^{-2} \cdot$hr^{-1}, and $k_+ = 3.69 \cdot 10^4$ M$^{-1} \cdot$hr^{-1} for the data shown above.

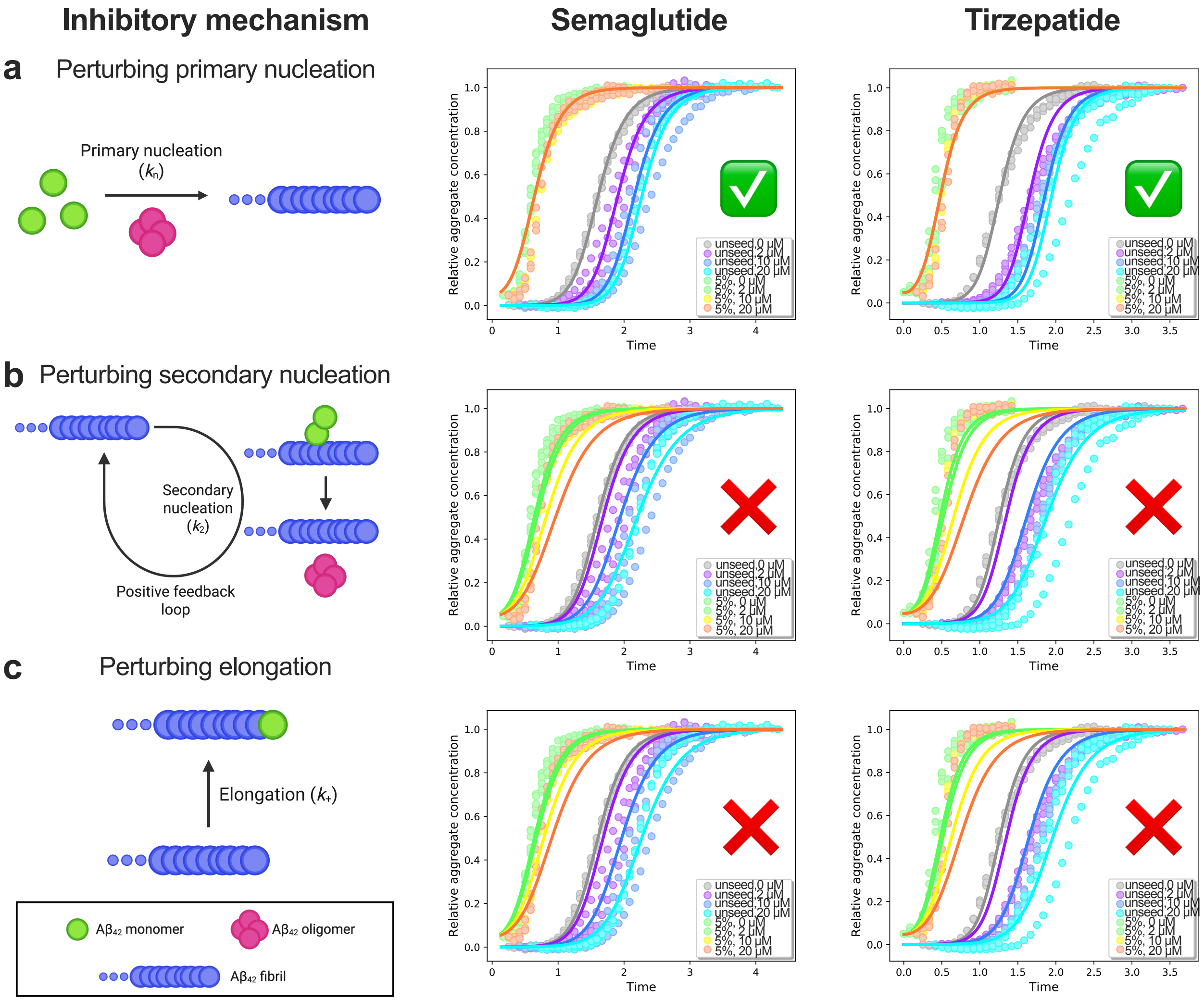

Figure S9: Kinetic fitting of the Aβ$_{42}$ aggregation traces in the presence of semaglutide and tirzepatide indicates a decrease in k_n, but not k_2 or k_+. Kinetic profiles of the aggregation of 2 µM Aβ$_{42}$ in the absence (grey) or presence of semaglutide (left panels) or tirzepatide (right panels) at molar ratios of Aβ$_{42}$ to GLP-1RA of 1:1, 1:5, and 1:10 (purple to light blue) and kinetic profiles of the aggregation of 2 µM Aβ$_{42}$ in the presence of 5% seeds in the absence (green) or presence of semaglutide or tirzepatide at molar ratios of Aβ$_{42}$ to GLP-1RA of 1:1, 1:5, and 1:10 (light green to orange). The unseeded and 5% seeded data were fit globally for an effect on (**a**) primary nucleation (k_n), (**b**) secondary nucleation (k_2), or (**c**) elongation (k_+). Solid lines indicate theoretical predictions based on kinetic fitting (see Results). Green checks indicate high-quality fits. Red X marks indicate misfits where the model fails to recapitulate the experimental data. Illustrations created with BioRender.com. Three technical replicates are shown for each condition. Data are representative of at least three independent experiments.

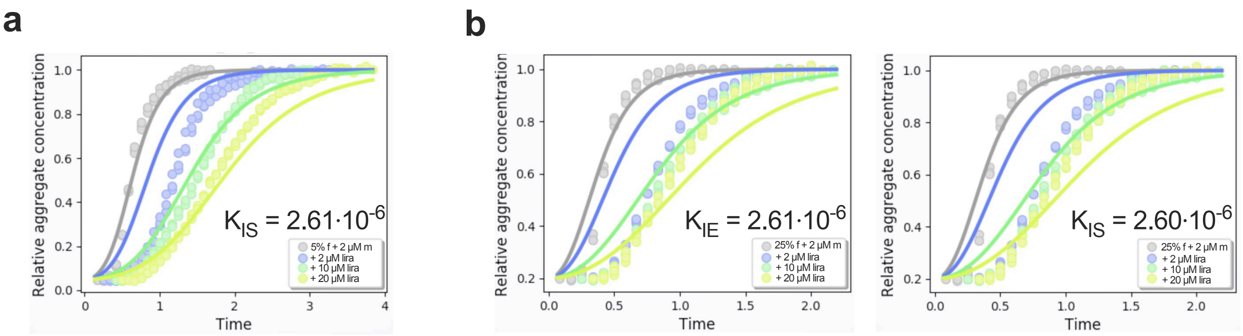

Figure S10: Seeding experiments confirm liraglutide has an impact on fibril-dependent microscopic processes in Aβ_{42} aggregation. 2 μM Aβ_{42} (in 20 mM sodium phosphate, 0.2 mM EDTA, 1% v/v DMSO, pH 8.0, quiescent) was aggregated in the presence of 5% (**a**) or 25% (**b**) fibril seeds and in the absence (grey) or presence of 2 (blue), 10 (green), or 20 μM (yellow) liraglutide. k_n, k_2, and k_+ were set as global constants using the procedure outlined in **Figure S8**. Data in (a) were then globally fit for perturbation to secondary nucleation only, which yielded decent fits with K_{IS} = 2.61 μM. Data in (b) were globally fit for perturbation to elongation only (left) or secondary nucleation only (right), which yielded decent fits with K_{IE} = 2.61 μM or K_{IS} = 2.60 μM.

a

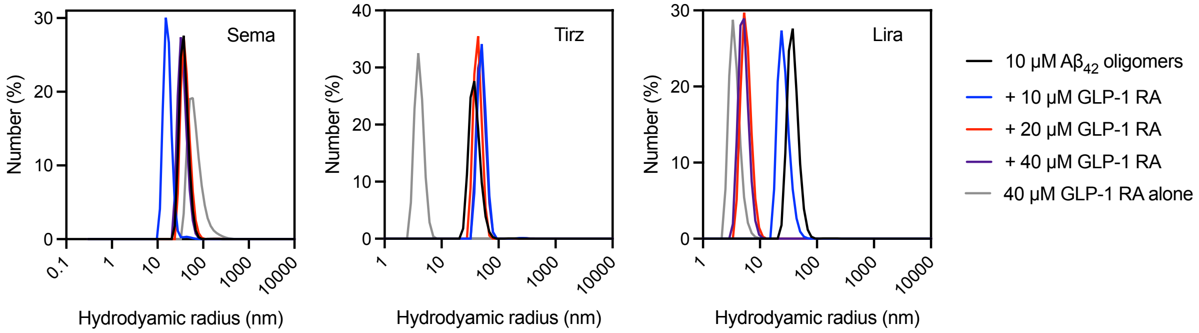

Figure S11. Dynamic light scattering measurements of stabilized Aβ_{42} oligomers exposed to GLP-1RAs. Aβ_{42} oligomers (black) at a concentration of 10 µM (in 20 mM sodium phosphate, 0.2 mM EDTA, pH 8.0, 2% v/v DMSO) were exposed for 1 h at 37 ˚C to 10 (blue), 20 (red), or 40 µM (purple) concentrations of semaglutide (left), tirzepatide (middle), or liraglutide (right). GLP-1RA alone controls at 40 µM are shown in grey. Oligomers exposed to semaglutide and tirzepatide were largely unchanged, but those exposed to liraglutide experienced a dose-dependent decrease in size. The effects of liraglutide were confirmed in two independent experiments from different oligomer preparations.

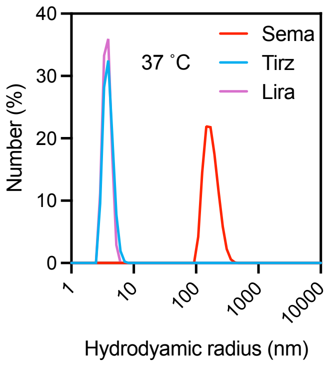

Figure S12: DLS of GLP-1RAs at 37 ˚C. Hydrodynamic radius measurements for 20 µM solutions of semaglutide (red), tirzepatide (blue), or liraglutide (purple) in 20 mM sodium phosphate, 0.2 mM EDTA, pH 8.0, 1% v/v DMSO.

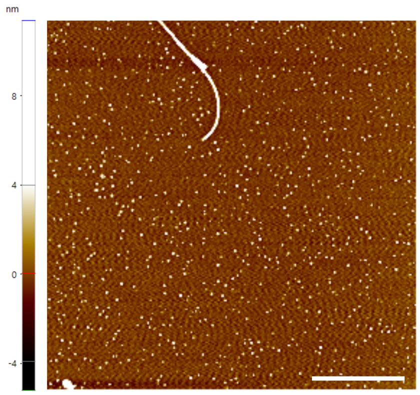

Figure S13: Presence of rare fibrils in AFM images for liraglutide alone. With extensive imaging, two occurrences of fibrillar species were observed in AFM maps for liraglutide incubated under the same conditions as in the other AFM images, but in the absence of Aβ_{42}. Scale bar, 1 μm.

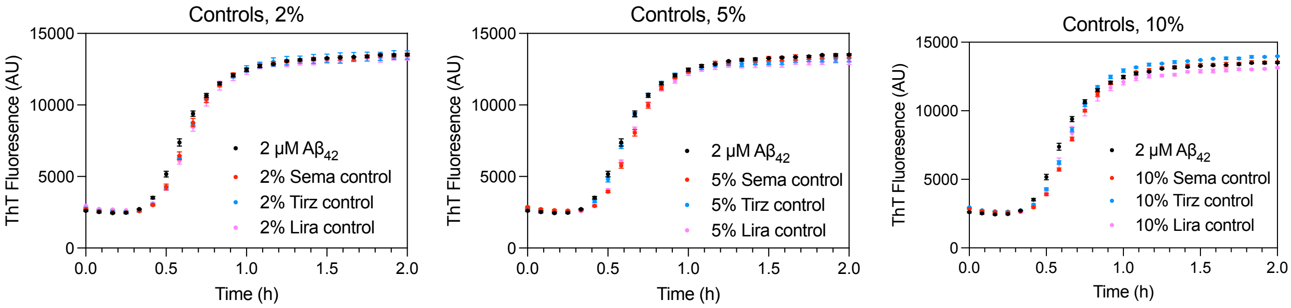

Figure S14: Control experiments for seeding capacity assays. Corresponding control stocks of GLP1-RAs prepared without $A\beta_{42}$ were centrifuged and resuspended twice to match the protocol in **Figure 6**. These stocks had no impact on $A\beta_{42}$ aggregation, indicating free GLP-1RA was not appreciably carried over in the sample preparation process.

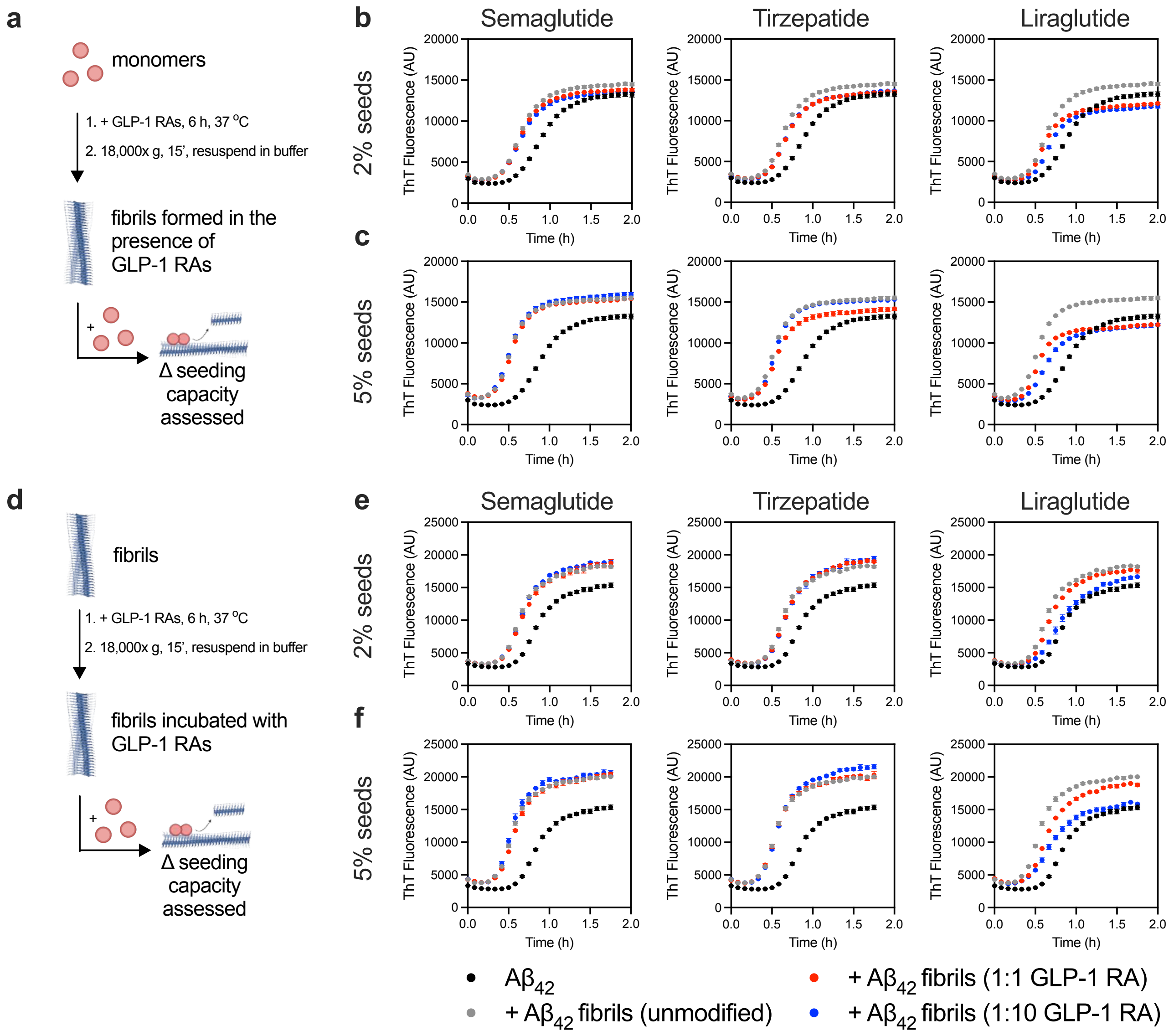

Figure S15: Liraglutide alone reduces the seeding capacity of Aβ42 fibrils. (**a**) 2 µM Aβ42 was aggregated to completion in the absence (black) or presence of 1:1 (red) or 1:10 (blue) molar ratios of Aβ42 to semaglutide, tirzepatide, or liraglutide. Samples were centrifuged to pellet insoluble material, then resuspended in phosphate buffer. Unmodified or GLP-1RA-modified fibril stocks were then used at 2% (**b**) or 5% (**c**) concentrations of seeds to catalyze the aggregation of 2 µM Aβ42. Fibrils formed with semaglutide or tirzepatide catalyzed Aβ42 aggregation similarly to unmodified fibrils, while those formed with liraglutide templated less effectively. (**d**) Pre-formed fibrils were incubated under the same conditions as (**a**), centrifuged, and resuspended in phosphate buffer. Those unmodified or GLP-1RA-modified fibril stocks were similarly used at 2% (**e**) or 5% (**f**) concentrations of seeds to catalyze the aggregation of 2 µM Aβ42. Again, only liraglutide reduced the templating ability of Aβ42 under these conditions. Error bars indicate s.e.m. of three technical replicates.

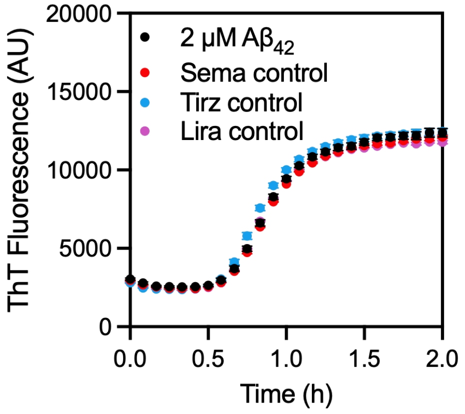

Figure S16: Seeding capacity control experiments. Samples containing the maximum tested concentrations of semaglutide (red), tirzepatide (blue), or liraglutide (purple) were prepared, incubated, centrifuged, and resuspended identically as in **Figure S15**. Since GLP-1RAs are soluble, we did not expect them to be retained by centrifugation. As expected, the aggregation of 2 μM $A\beta_{42}$ was not impacted when seeded with GLP-1RA alone stocks, indicating residual GLP-1RA is not appreciably transferred during sample preparation. The reduction in seeding capacity observed with liraglutide is not related to the presence of free GLP-1RA carried over during sample preparation.

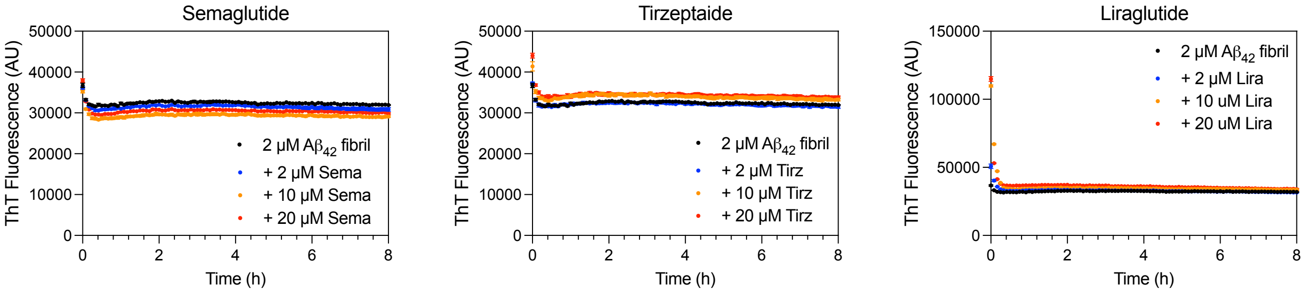

Figure S17: Semaglutide, tirzepatide, and liraglutide do not disaggregate Aβ_{42} fibrils. Samples containing 2 µM Aβ_{42} fibrils (20 mM sodium phosphate, 0.2 mM EDTA, 1% v/v DMSO, pH 8.0, quiescent), 20 µM ThT, and 0 (black), 2 (blue), 10 (orange), or 20 µM (red) of semaglutide (left), tirzepatide (middle), or liraglutide (right) were prepared on ice. Samples were then measured in triplicate at 37 ˚C. The ThT signal was stable over time, indicative of a lack of Aβ_{42} fibril disaggregation by GLP-1RAs.

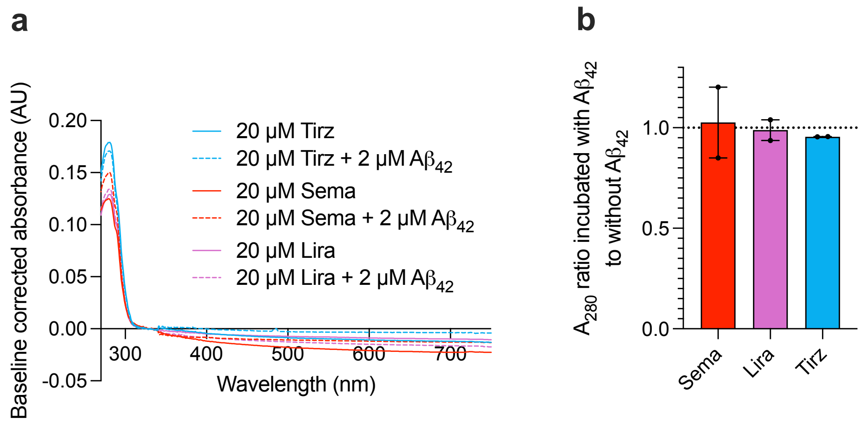

Figure S18: GLP-1RAs remain largely soluble during Aβ_{42} aggregation. (**a**) GLP-1RA solutions at 20 µM were incubated exactly as in the kinetic experiments with or without 2 µM Aβ_{42} until all samples had finished aggregating according to tracer samples containing ThT in adjacent wells (~6 h). Solutions were collected from multiwell plates, centrifuged at 18,000x g for 15 min, and supernatants carefully collected and measured using absorbance spectroscopy from 270 to 750 nm. Spectra that were baseline corrected at 330 nm, as shown, were highly similar when semaglutide, liraglutide, or tirzepatide were incubated with and without Aβ_{42}. (**b**) Quantification of A_{280} was largely unchanged by incubation of these GLP-1RAs with Aβ_{42}. Error bars indicate s.e.m. of two independent experiments.

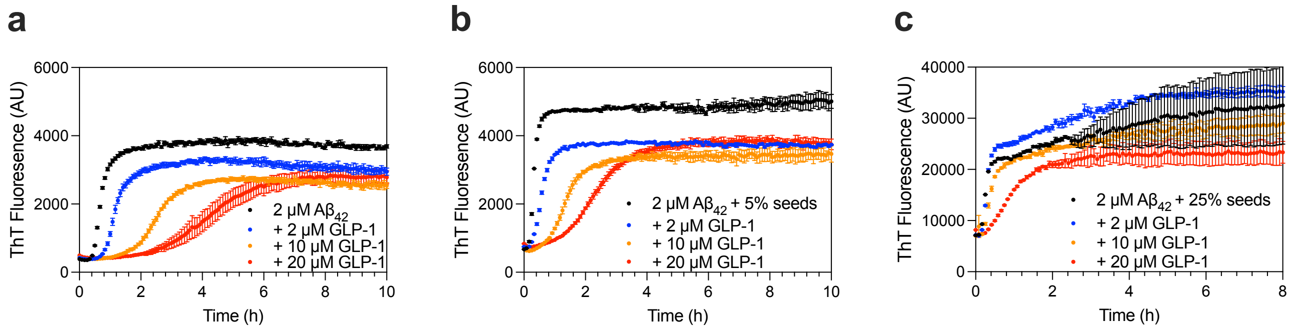

Figure S19: ThT assays for unseeded and seeded Aβ42 aggregation with GLP-1(7-37). Experiments were performed using 2 μM Aβ42 (in 20 mM sodium phosphate, 0.2 mM EDTA, 1% v/v DMSO, pH 8.0, quiescent) in the absence (black) or presence of 2 (blue), 10 (orange), or 20 μM (red) concentrations of GLP-1 with no fibril seeds (**a**), 5% fibril seeds added at the start of the aggregation reaction (**b**), or 25% fibril seeds added at the start of the aggregation reaction (**c**). Normalized data are shown and modeled in **Figure S20**. Error bars indicate s.e.m. of three technical replicates. Data are representative of two independent experiments.

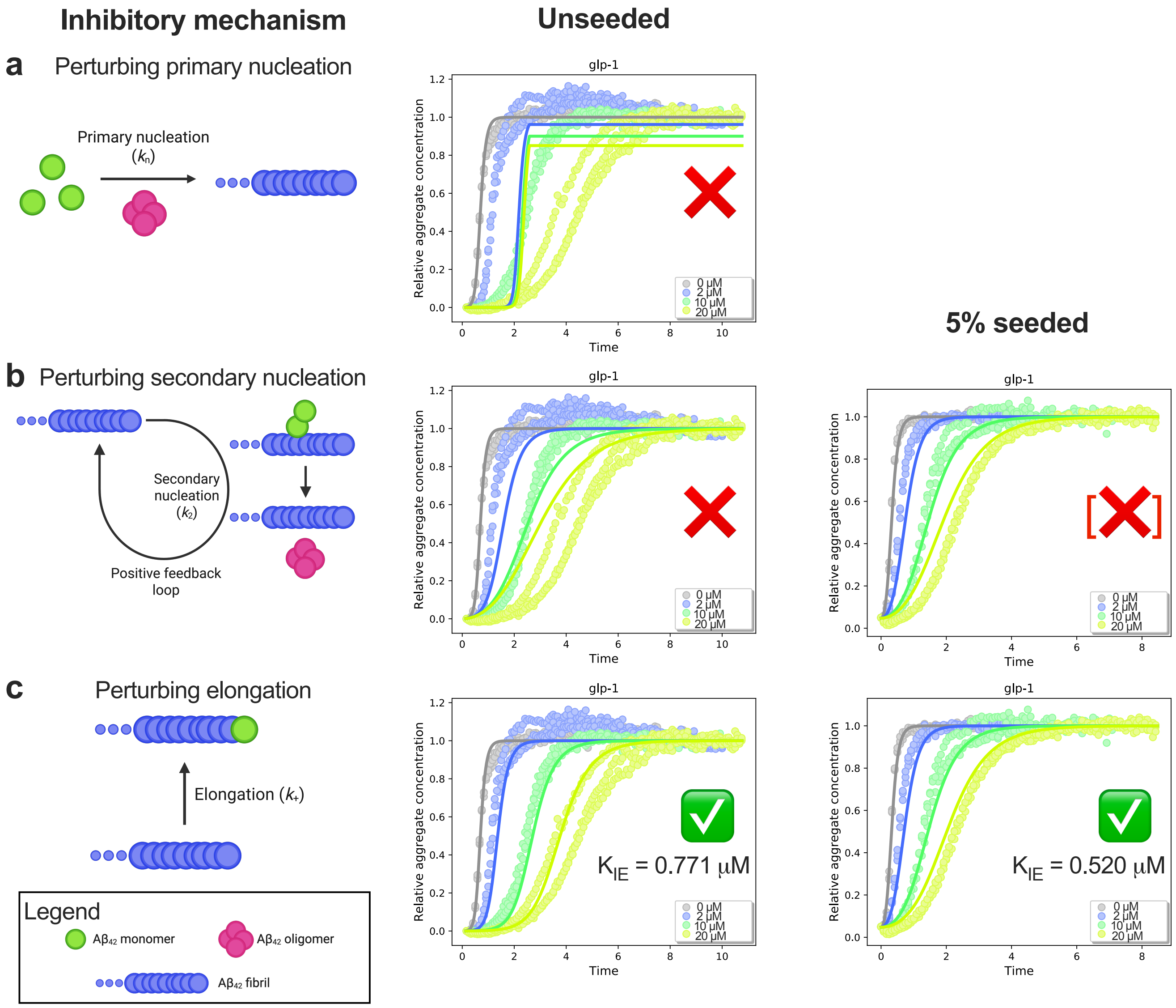

Figure S20: GLP-1(7-37) inhibits Aβ42 aggregation by suppressing elongation. Mechanism of action determination for GLP-1(7-37) by global fitting Aβ42 aggregation kinetics with different concentrations of GLP-1(7-37). Experiments were performed using 2 μM Aβ42 (in 20 mM sodium phosphate, 0.2 mM EDTA, 1% v/v DMSO, pH 8.0, quiescent) in the absence (grey) or presence of 2 (blue), 10 (green), or 20 μM (yellow) concentrations of GLP-1(7-37) with no seeds (middle panels) or 5% seeds (right panels). Solid lines indicate theoretical predictions based on kinetic fitting (see Results). During fitting, the rate constants for primary nucleation (k_n), secondary nucleation (k_2), and elongation (k_+) were determined for Aβ42 alone, and then held as global constants while fitting for perturbations to only (**a**) primary nucleation, (**b**) secondary nucleation, or (**c**) elongation. Green checks indicate excellent fits with associated IC_{50} values for elongation (K_{IE}). Red X marks indicate misfits where the model fails to recapitulate experimental data. The [X] symbol indicates lower quality fits. Illustrations created with BioRender.com. Three technical replicates are shown for each condition.

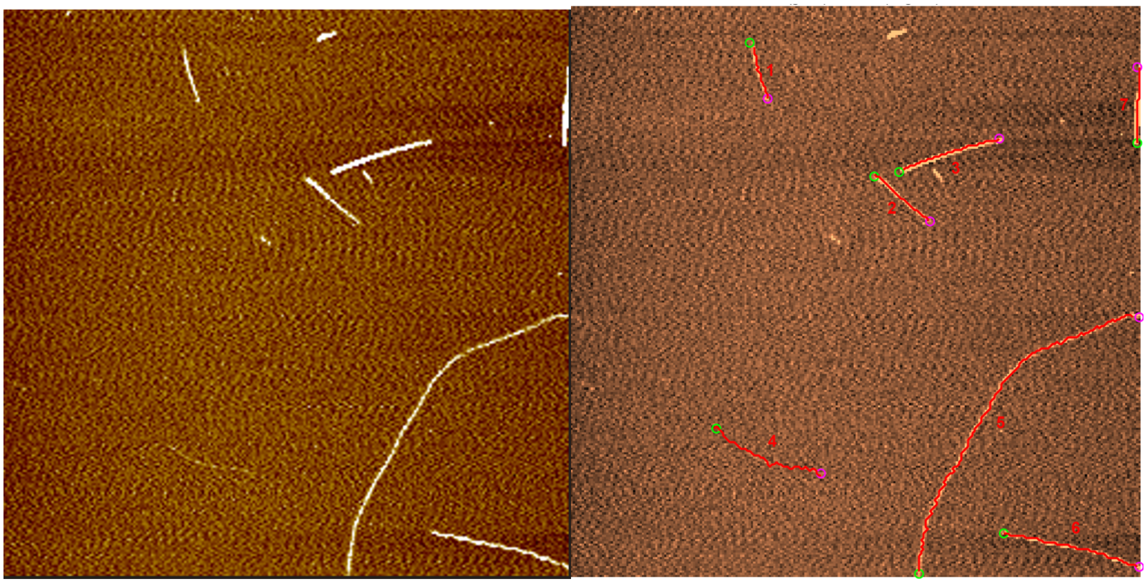

Figure S21: Representative AFM images showing fibril analyses. AFM maps were either 4 µm × 4 µm or 10 µm × 10 µm 10-bit TIFF images with a lateral step size (pixel width) of 3.9 or 9.8 nm, respectively. Manually selected fibril endpoints (green and magenta circles) defined each fibril on AFM height maps. A custom MATLAB code traced the fibril backbone (red line) between each pair of green/magenta endpoints using the bskel function and svgolay smoothing filter (11-point window) functions. Fibril were labeled (e.g., numbered 1 through 7 in the right panel) to correlate individual descriptive statistics. Ten replicate trials of an entire AFM map confirmed the reproducibility of this approach. The minimum fibril length was 75 nm. The relative uncertainty in tortuosity and fibril length was 2% and <1%, respectively. Reported tortuosity and length values are based on 212, 98, 214, and 69 fibrils for $A\beta_{42}$, $A\beta_{42}$ + Sema, $A\beta_{42}$ + Tirz, and $A\beta_{42}$ + Lira, respectively. Pathlength values are based on the subset of interior fibrils with both endpoints visible within the AFM map.

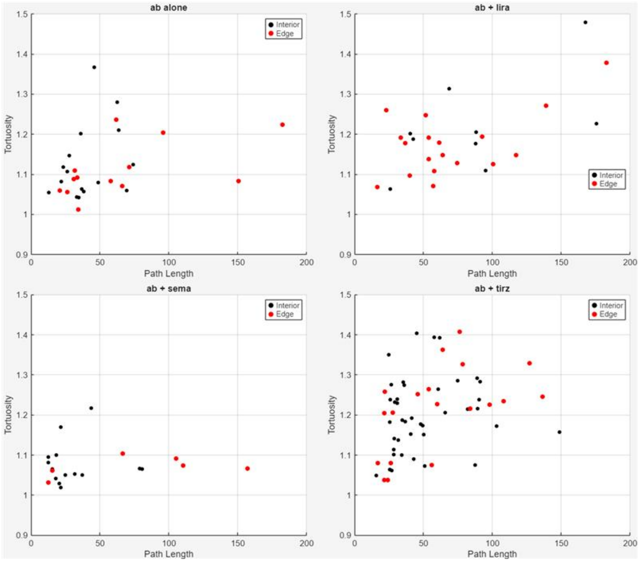

Figure S22: Fibril path length analysis. Comparison of interior and edge fibrils (endpoints within three pixels of a map boundary) indicated no significant difference in tortuosity among each fibril set, so edge-terminating fibrils were included in the final tortuosity analyses.
